# Enhancing the promiscuity of a member of the Caspase protease family by rational design

**DOI:** 10.1002/prot.25950

**Published:** 2020-06-11

**Authors:** Christoph Öhlknecht, Drazen Petrov, Petra Engele, Christina Kröß, Bernhard Sprenger, Andreas Fischer, Nico Lingg, Rainer Schneider, Chris Oostenbrink

**Affiliations:** ^1^ Institute of Molecular Modeling and Simulation University of Natural Resources and Life Sciences Vienna Austria; ^2^ Institute of Biochemistry and Center of Molecular Biosciences Innsbruck University of Innsbruck Innsbruck Austria; ^3^ Austrian Centre of Industrial Biotechnology Vienna Austria

**Keywords:** free‐energy calculations, fusion proteins, in‐silico modeling, molecular simulation, promiscuity, proteases

## Abstract

The *N‐*terminal cleavage of fusion tags to restore the native *N‐*terminus of recombinant proteins is a challenging task and up to today, protocols need to be optimized for different proteins individually. Within this work, we present a novel protease that was designed in‐silico to yield enhanced promiscuity toward different *N‐*terminal amino acids. Two mutations in the active‐site amino acids of human Caspase‐2 were determined to increase the recognition of branched amino‐acids, which show only poor binding capabilities in the unmutated protease. These mutations were determined by sequential and structural comparisons of Caspase‐2 and Caspase‐3 and their effect was additionally predicted using free‐energy calculations. The two mutants proposed in the in‐silico studies were expressed and in‐vitro experiments confirmed the simulation results. Both mutants showed not only enhanced activities toward branched amino acids, but also smaller, unbranched amino acids. We believe that the created mutants constitute an important step toward generalized procedures to restore original *N‐*termini of recombinant fusion proteins.

## 1 INTRODUCTION

Within the last decades, the advent of recombinant protein production has changed the manufacturing processes of pharmaceutical industry significantly. The first recombinant pharmaceutical that was used succesfully for treatment of humans was insulin in 1982.^1^ With recent advances in modern molecular biology techniques, recombinant protein technologies have become one of the mainstream methods for production of pharmaceutical proteins. Modern recombinant proteins used for clinical treatments include hormones, interferones, interleukines, growth factors, tumor necrosis factors, blood clotting factors, thrombolytic drugs, and various enzymes for the treatment of major diseases like—to mention only a few—diabetes, congestive heart failure, multiple sclerosis, anemia, hepatitis, asthma, or cancer.^2^

Recombinant proteins are typically produced in host cells of bacterial, fungal, or animal origin. Usually, cells are separated from the product‐containing cell culture media by centrifugation or filtration. The protein of interest is then separated from the crude media solution using chromatographic purification.^3^ In order to obtain a highly purified product, usually multiple chromatographic purification steps are performed sequentially,^4^, ^5^ which consequencently raises the production costs significantly.

To enhance the purification process and lower the costs, affinity tags can be used.^6^, ^7^, ^8^ These get linked to the recombinant protein, resulting in a so‐called fusion protein, which shows modified chromatographic properties. However, fusion‐tags can influence the structure and characteristics of the protein and may even cause immune reactions when used for in‐vivo treatment.^9^ Therefore, after succesful purification, the native *N‐* or *C‐* terminus has to be restored when the recombinant protein is intended to be used for therapeutic applications. Thus, fusion proteins are usually equipped with a specific cleavage site that can be targeted for chemical or enzymatic hydrolysis.^10^, ^11^, ^12^, ^13^, ^14^

Up to this day there is no universal procedure to cleave fusion tags from a wide variety of proteins. One reason is that *N‐* and *C‐*terminal protein sites vary in their physical and chemical properties. Hence, several procedures exist for tag removal. A very common tool are endoproteases. Serine proteases such as factor Xa,^15^ *α*‐thrombin,^16^ or enterokinase^17^ have high turnover rates but rather low specificity attributed to them.^18^ Viral proteases like tobacco etech virus protease (TEV)^19^ or human rhinovirus 3C protease,^20^ however, are more specific at lower turnover numbers.^18^, ^21^ In recent years, self‐cleaving enzymes like Inteins^22^, ^23^ have attracted interest.^24^ But there are still too many disadvantages to using inteins for them to be implemented on a large scale: premature cleavage,^21^ slow cleavage kinetics,^25^, ^26^ need for a high effective concentration,^23^ too high expression load due to large size.^27^ From this list of possible systems, we are able to deduce that cleavage protocols are far from universal, but have to be optimized specifically for individual proteins or tags. Therefore, a standardized enzymatic or chemical procedure for processing a large amount of recombinant fusion proteins is highly desirable. It needs to be highly unspecific toward the terminus of the protein that is bound to the tag, but at the same time specific to the cleavage site of the tag, to minimize off‐target effects, possibly rendering the recombinant protein unusable for pharmaceutical applications. This could be a protease that is highly specific in recognizing potential binding sites in one terminal direction but is rather promiscuous in the other terminal direction from the site of cleavage.

**FIGURE 1:**
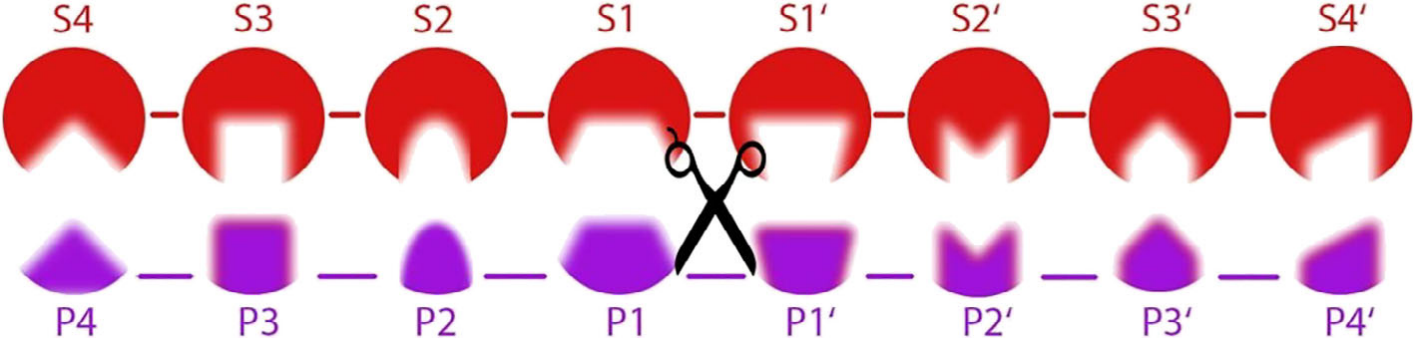
The Schechter and Berger protease nomenclature for protease‐substrate binding.^28^ The protease subpockets are denoted with an S while the substrate binding sites are denoted with a P. The protease subsites and the substrate binding sites in *C‐*terminal direction from the site of cleavage are marked with a single prime symbol. The site of hydrolysis is the linkage between P1 and P1', as indicated by the scissor [Color figure can be viewed at wileyonlinelibrary.com]

This problem can be discussed best using the Schechter and Berger protease nomenclature (Figure 1).^28^ The advantage of this scheme is that each binding site can be assigned a selectivity measure individually. Such selectivity measures can be obtained by statistical analysis of substrate data that was derived by experimental screening methods, as available in the MEROPS database.^29^

**FIGURE 2:**
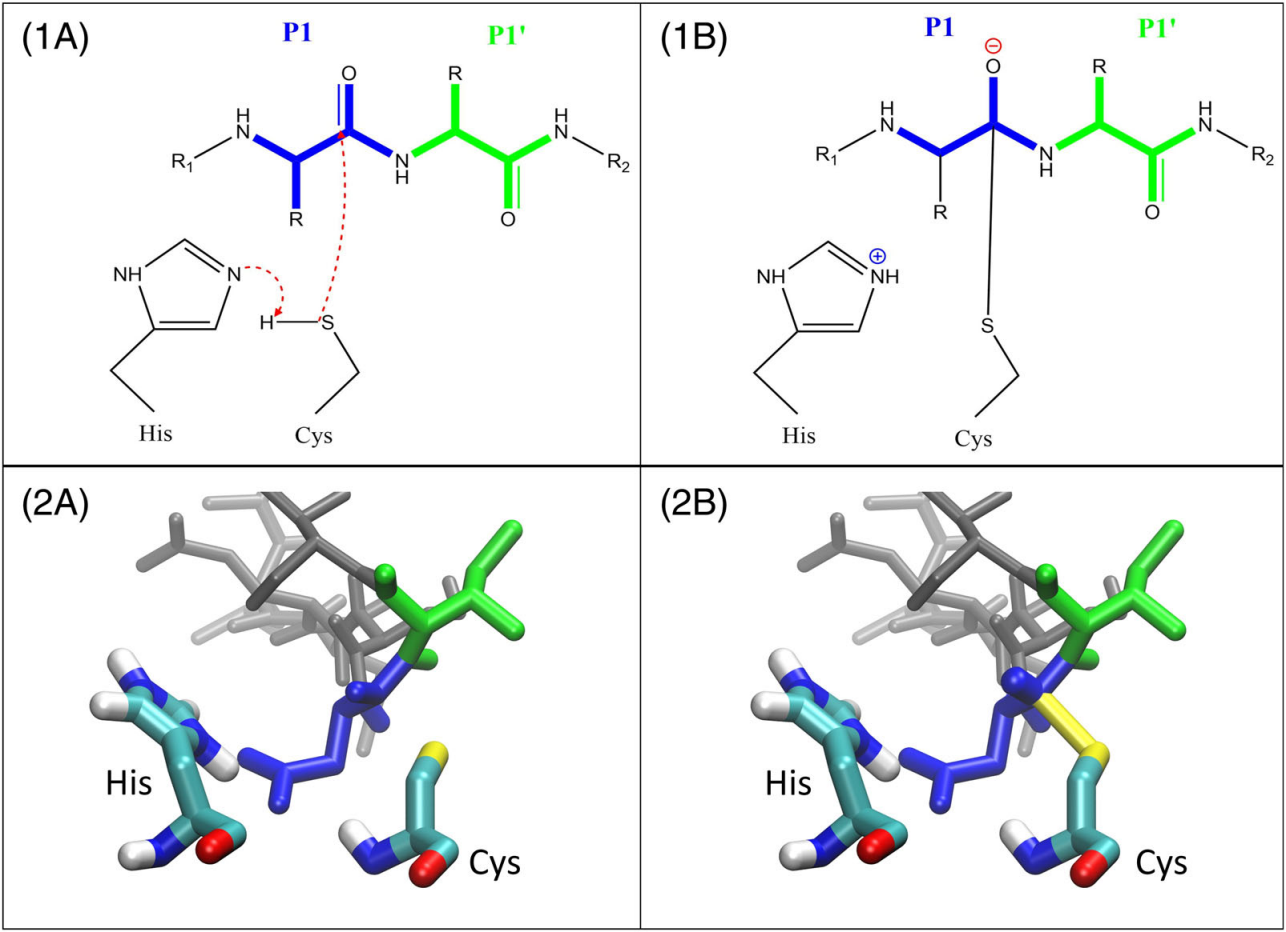
First step of the caspase reaction mechanism. 1.A, The base His277 activates Cys220 which then undergoes a nucleophilic attack on the carbonyl group of the P1 amino acid. 2.A, A snapshot of the active site visualising the noncovalently bound state. P1 amino acid (Asp) is shown in blue and P1' amino acid (Ile) is shown in green. 1.B, The reactive cysteine has formed a bond with the respective carbonyl group, a tetrahedral intermediate state has formed. 2.B, The same snapshot as in 2.A is shown. A bond was inserted between Cys220 and the carbonyl group of the P1 amino acid [Color figure can be viewed at wileyonlinelibrary.com]

In the current work, human Caspase‐2 was designed toward the need for a biochemical scissor to generate natural *N‐*termini of recombinant fusion proteins. Caspases (cysteine‐dependent aspartate‐directed proteases) are a family of protease enzymes and are the most important inducers of cell apoptosis in animals.^30^ They are named according to their specific mode of hydrolysis. A cysteine first gets activated by a histidine, and then nucleophilically attacks the carbonyl C‐atom of the protein backbone after an aspartate residue in the target protein sequence (Figure 2). Twelve different members of the Caspase family have been described in humans.^31^, ^32^ Caspases are abundant in all animals and are even related to plant and fungal analogous proteases called metacaspases.^33^

Caspase‐2 was selected as a starting basis for further design since it specifically recognizes and binds to a pentapeptide in the *N‐*terminal direction from the site of cleavage (P1‐P5) rather than a tetrapeptide (P1‐P4), as bound by other caspases.^34^ Catalytic studies have found that Caspase‐2 has a more than 1000‐fold lower *k*_cat_/*K*_*M*_ value toward tetrapeptides compared to Casp‐1 and Caspase‐3.^35^ However, pentapeptides have been shown to be cleaved by Caspase‐2 with a tenfold higher efficiency than tetrapeptides, indicating that this caspase has a more extended binding pocket.^36^ In summary, Caspase‐2 is a less efficient catalyst compared to other caspases (like Caspase‐3) for the benefit of higher specificity. Unfortunately, in Caspase‐2 the binding pockets in the *C‐*terminal direction from the site of cleavage are not entirely unspecific. Specifically, the S1' site has a major contribution in the substrate recognition process as it selectively and preferentially recognizes glycine, serine and alanine over the other amino acids.^32^ The P1' site corresponds to the *N‐*terminal amino acid of the recombinant protein, so promiscuity at this site is of major importance. Thus, the motivation of the underlying work was to modify the active site such that the S1' site becomes more promiscuous toward different amino acids, while keeping the selectivity of the S1 to S5 subsites as high as possible.

Changing the specificity of proteases is a problem that was researched quite extensively in recent decades. Successes were achieved in various disciplines with directed evolution approaches being highly successful methods. This is due to high‐throughput assay systems having become available.^37^ Two big advantages of directed evolution are that it can be done succesfully without any information about the protein structure. Additionally, effective mutations at positions in distance from the actual binding site can be found. Great successes have however, also been achieved by rationally redesigning the individual binding pockets by alternating polarity and packing.^38^, ^39^, ^40^ In the current work, a rational design approach is described. First, the promiscuities across all members of the human caspase family were analyzed by comparing statistical cleavage data. Further structural analysis together with free energy‐calculations were performed to screen the mutants computationally. For alchemical perturbations involving net‐charge changes of the system (as one encounters when perturbing a noncharged amino acid into an amino acid with a charged side chain), the charging free energies were corrected by calculating the methodology‐dependent error using continuum electrostatics models.^41^, ^42^, ^43^ As a result, two protein mutants were proposed that were calculated to be better binders of substrates hosting isoleucine at the P1' site and thus were believed to show better binding toward apolar or polar, branched amino acids. To validate the computational predictions and to test the effect of the proposed mutations, the influences of P1' on cleavage relative to P1' Gly were measured experimentally, both in the mutants and the unmutated protein.

The kinetics and binding were studied in more detail for selected substrates by determining Michaelis‐Menten kinetic parameters. Förster Resonance Energy Transfer (FRET) is an effect that can be used to determine the kinetics of a protease catalyzed reaction.^34^ A peptide carrying a fluorophore such as 2‐aminobenzoyl (Abz) and a quencher such as 2,4‐dinitrophenyl (Dnp) will exhibit only a low level of fluorescence. Upon cleavage of the peptide with an appropriate protease, the fluorescence signal will increase proportionally to the change in concentration. This allows for online monitoring of the cleavage reaction, the initial slope of which can then be fitted to the Michaelis‐Menten equation.

## 2 METHODS

### 2.1 Statistical analysis

Data for a statistical analysis of known substrates of Caspase‐1, ‐2, ‐3, ‐6, ‐7, and ‐8 was downloaded from the Merops Database.^29^ The data was normalized by the natural occurrence of the amino acids in humans (as found in the UniProt Knowledgebase^44^). For comparisons between different subsites and various proteins, statistical cleavage data was quantitatively expressed in terms of subsite‐specific cleavage entropies^45^:

(1) $$S_{i}=-\sum_{a=1}^{20}p_{a,i}\cdot^{20}\log p_{a,i}$$

where *p*_*a*,*i*_ denotes the (normalized) probability of occurrence of amino acid *a* in subsite *i*. The quantity *S*_*i*_ is dimensionless and defined such that *S*_*i*_ = 0 for a perfectly specific pocket *i* (only allowing for one amino acid to bind) and *S*_*i*_ = 1 for a uniform probability distribution over all amino acids.

### 2.2 Sequence analysis

To evaluate if Caspase‐3 could serve as a possible model protein for engineering the Caspase‐2 active site, the sequences between the two proteins were aligned using the Needleman‐Wunsch algorithm.^46^ This was done for the amino acid sequence of the entire protein and the amino acid sequences of the active site regions only. The relevant amino acid regions were gathered by structural visualization of the two proteins using the VMD program.^47^ Roughly, contiguous sequences of 35 amino acids hat were at most 1 nm removed from the P1' residue were selected.

### 2.3 MD simulations

The Caspase‐2 crystal structure in complex with the inhibitor *N*‐acetyl‐l‐leucyl‐l‐*α*‐aspartyl‐l‐*α*‐glutamyl‐l‐seryl‐l‐aspartic aldehyde (PDB ID:1PYO^48^) were retrieved from the PDB data bank (http://www.rcsb.org).^49^ Caspase‐2 was resolved as a functional dimer, with a disulfide bridge linking the two monomers. The inhibitor was extended after the C‐terminus by a chain of the sequence Ile‐Val‐Ser‐Ser to span the entire active site using the MOE 2017 loop modeler.^50^ Three representative substrate starting structures were chosen. Subsequently, 120 ns long molecular dynamics (MD) simulations (40 ns per substrate starting structure) were performed. The last 30 ns of the individual simulations were used to find a representative substrate starting structure for the subsequent free‐energy calculations. This was done by clustering using the algorithm by Daura et al^51^ with a cutoff distance of 0.23 nm. One representative structure was chosen from the dominant cluster. All simulations were run using the GROMOS11 molecular simulation software (http://www.gromos.net).^52^

Interactions between proteins, substrates and other constituents of the studied systems were described with the GROMOS 54A8 parameter set.^53^ In order to focus on configurations that are relevant in the actual substrate cleavage process, the sampling of the protein‐substrate complex was initially based on a description of the tetrahedral intermediate state (Figure 2).^54^ This intermediate state was modelled by covalently linking the substrate to the active site cystein. The parameters for this intermediate state were generated using the Automated Topology Builder (http://atb.uq.edu.au/),^55^, ^56^, ^57^ and made available in Figure S5 and Data S1.

Hydrogen atoms were added to the starting structure according to geometric criteria and energy was minimized using the steepest‐descent algorithm. For all simulations, water was treated explicitly and implemented by means of the three‐site simple point charge (SPC) model.^58^ Simulations were carried out under periodic boundary conditions (PBC) based on rectangular computational boxes with at least 0.8 nm between any protein atom and the nearest box wall. The equations of motion were integrated using the leap‐frog scheme.^59^ Bond vibrations were constrained using the SHAKE algorithm^60^ with a relative geometric tolerance of 10^−4^. The centre of mass translation of the computational box was removed every 2 ps. The temperature and pressure were maintained at 298.15 K and 1 atm by weak coupling^61^ using a coupling time of *τ*_*T*_ = 0.1 ps and *τ*_*P*_ = 0.5 ps and an isothermal compressibility of 7.624 × 10^−4^(kJ mol^−1^ nm^−3^)^−1^.^61^ Electrostatic interactions were calculated using a Barker‐Watts reaction field (BM) scheme^62^ with a value *ε*_*BW*_ = 61. Nonbonded interactions were calculated using a molecular twin‐range charge‐group cutoff scheme. The cut‐off used for the short‐range pairlist construction was set to 0.8 nm and the cut‐off used for the long‐range interactions was set to 1.4 nm. Interactions within the short range were calculated every time step from a pairlist that was updated every 10 fs. At pairlist updates interactions up to the long‐range cutoff were computed and kept constant.

### 2.4 Free‐energy calculations

**FIGURE 3:**
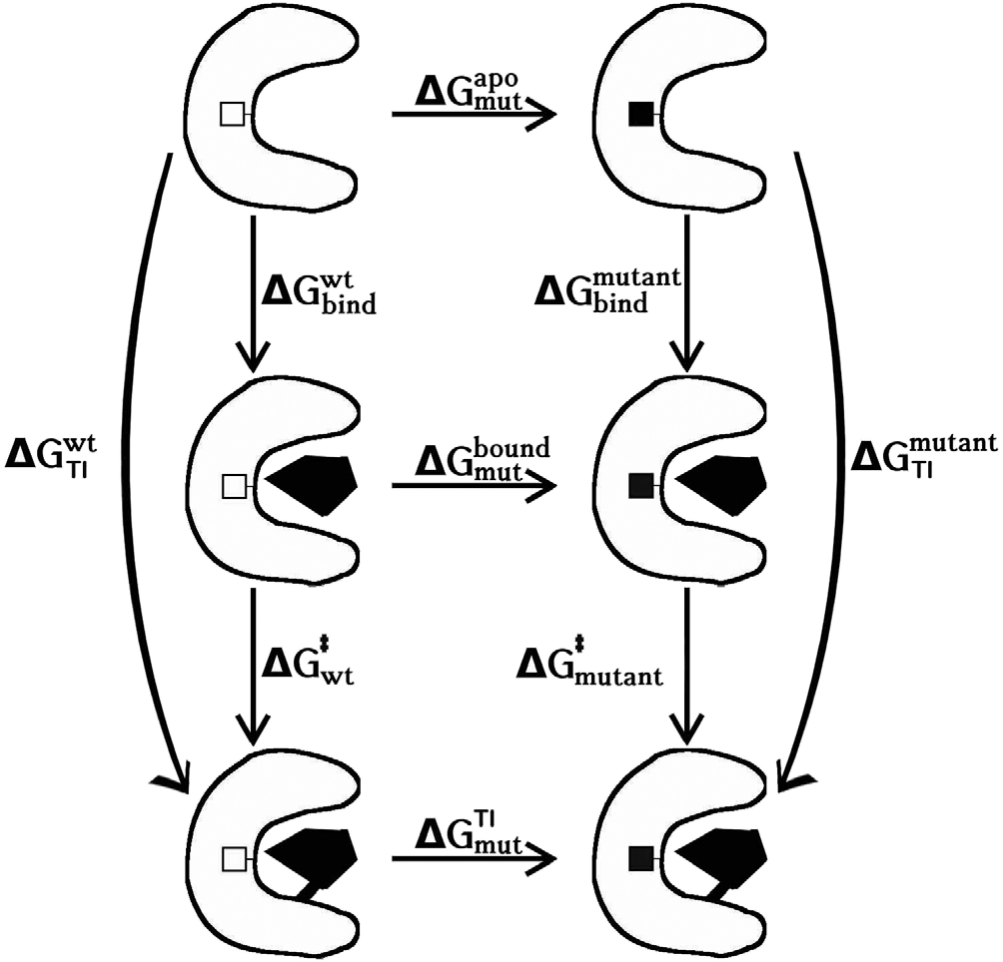
Thermodynamic cycle to model protein mutations. The horizontal arrows represent alchemical mutations. The vertical arrows represent the binding free energies. The differences for the physical binding processes, $\Delta \Delta G_{\text{bind}}=\Delta G_{\text{bind}}^{\text{mutant}}-\Delta G_{\text{bind}}^{\mathrm{wt}}$ and $\Delta \Delta G_{\mathrm{TI}}=\Delta G_{\mathrm{TI}}^{\text{mutant}}-\Delta G_{\mathrm{TI}}^{\mathrm{wt}}$ were calculated from the alchemical free‐energy estimates, $\Delta \Delta G_{\text{bind}}=\Delta G_{\mathrm{mut}}^{\text{bound}}-\Delta G_{\mathrm{mut}}^{\mathrm{apo}}$ and $\Delta \Delta G_{\mathrm{TI}}=\Delta G_{\mathrm{mut}}^{\mathrm{TI}}-\Delta G_{\mathrm{mut}}^{\mathrm{apo}}$. Analogously, the difference for the catalytic step was calculated as $\Delta \Delta G^{‡}=\Delta G_{\mathrm{mut}}^{\mathrm{TI}}-\Delta G_{\mathrm{mut}}^{\text{bound}}$

All changes in protein‐substrate binding free energies were calculated along thermodynamic cycles in which the mutations were modelled alchemically twice: alchemical changes of the protein were modelled both in the protein‐substrate complex (bound structure) and in the protein without a substrate (apo structure) (Figure 3); alchemical changes of the substrate were modelled in the protein‐substrate complexes of the mutant and the unmutated Caspase‐2. In total, three sets of free‐energy calculations were performed: the main free‐energy calculations were performed with the unbound (apo) protein and the covalently bound protein‐substrate complex in the tetrahedral intermediate state. These free energies correspond to the free‐energy differences between the intermediate state and the unbound state, with neglect of the actual binding process. To also capture the latter energies, additional calculations were performed with the substrate noncovalently bound, that is, according to Figure 2a. These calculations were only performed on mutations that were found to be favorable in the calculations of the tetrahedral intermediate. A final set of free‐energy calculations was performed on the P1‐P5 sites of the substrate rather than on the protein itself. Here, the amino acids of these five sites were perturbed into Ala individually (keeping all four other substrate binding sites unperturbed). These free‐energy calculations were performed in the fully mutated protein and in the unmutated protein to assess possible alternations in selectivity of the S1 to S5 subsites.

Changes in binding free energies (Δ*G*_*A* → *B*_) were calculated with the thermodynamic integration (TI) approach^63^ along progressive perturbations using a *λ*‐dependent Hamiltonian of the system, according to

(2) $$\Delta G_{A\to B}=\int_{0}^{1}\frac{\mathrm{dG}\left(\lambda \right)}{\mathrm{d\lambda}}\mathrm{d\lambda}=\int_{0}^{1}{〈\frac{\partial H\left(\lambda \right)}{\partial \lambda }〉}_{\lambda }\mathrm{d\lambda}$$

where *λ* denotes the scaling parameter of the TI procedure and 〈…〉_*λ*_ denotes ensemble averaging over configurations sampled at a given value of *λ*. The property $\frac{\partial H\left(\lambda \right)}{\partial \lambda }$ was written out every 40 fs during the simulation. From simulated *λ*‐points (max. 11 per perturbation), further *λ*‐points were predicted, yielding 101 *λ*‐points in total using the extended thermodynamic integration procedure.^64^ Every mutation was performed twice in the dimeric protein structures. Standard deviations of the free‐energy differences were calculated from the three independent simulations and the two active sites. After 11 equidistant *λ*‐values were simulated for 10 ns per *λ*‐value initially, the termodynamic integration profiles were refined by prolonging simulations up to 50 ns to bring the error‐estimates down.

### 2.5 Corrections for charging free energies

Charging free energies, as calculated for a perturbation of a noncharged amino acid into a charged amino acid (or vice versa), are typically very sensitive to the employed simulation methodology.^41^, ^65^, ^66^, ^67^ Because electrostatic energies are usually calculated using non‐Coulombic interaction functions (eg, lattice summation^68^, ^69^ or BW method^62^) under PBC, the calculated electrostatic potentials deviate from the “real” potentials. If only partial charges are perturbed and the (total) net charge of a set of atoms (eg, a charge group or a molecule) stays the same, these errors, that stem from the differences in the electrostatic potentials, mostly cancel. But perturbing a noncharged group of atoms into a charged group of atoms (or vice versa) is similar to the situation as solvating an ion, particularly bringing it from vacuum into solvent. In this situation, the inaccurate electrostatic potentials directly affect the calculated charging free energies. Hence, these quantities must be corrected ex post in order to achieve methodological independence. In short, the “correct” charging free energies can be calculated using continuum electrostatics methods and analytical models. These corrections must account for (a) the deviation of the solvent polarization around the charged group of atoms due to the use of a microscopic system in combination with cutoff‐truncation and a reaction‐field correction, relative to the “correct” polarization in a macroscopic, nonperiodic and fully Coulombic environment, (b) the deviation of the solvent‐generated electric potential in a microscopic box under PBC, relative to the “correct” potential under full Coulombic, macroscopic and non‐PBC, (c) the inaccurate electrostatic interactions between the charged group of atoms and other solute atoms due to the usage of cutoff‐truncation in combination with a reaction field correction, and (d) an inaccurate dielectric permittivity of the employed solvent model.^42^, ^70^ Note, that the correction terms (a), (b), and (c) were called Δ*G*_pol_, Δ*G*_psum_, and Δ*G*_dir_ in Ref. [42], while correction term (d) was not listed explicitly there, since this term is typically relatively small and was included in the Δ*G*_pol_ correction.^43^

### 2.6 Protein expression

Wildtype Caspase‐2 has to be activated by autocleavage.^71^ To express a variant of Caspase‐2 that is fully active without proteolytic cleavage, an uncleavable, circularly permuted variant of Caspase‐2 was generated.^72^, ^73^, ^74^ It is noted here that for all experimental tests, circularly permuted Caspase‐2 was used, for the sake of simplicity, we keep the term Casp‐2 for the circularly permuted version throughout the entire work.

For expression the Casp‐2 construct was transformed into BL21(DE3) cells. An overnight culture in TB medium (1.2% peptone, 2.4% yeast extract, 0.4% glycerol, 17 mM KH_2_PO_4_, and 72 mM K_2_HPO_4_) was incubated on a shaker at 37°C and 220 rpm for 16 hours. The preculture was diluted 1:100 in TB medium. The culture was induced with 0.4 mM IPTG at OD_600_ = 1.2 and 25°C. Four hours after induction, the cells were harvested by centrifugation at 3273*g* at 4°C for 30 min and stored at −20°C until purification.

For purification, the frozen cell pellets were suspended in Tris‐Buffer (50 mM Tris, 50 mM NaCl, pH 7.5) before disruption with a French press. The clarified supernatant was applied to an IMAC column (HisTrap FF Crude, 1 mL, GE Healthcare). After washing with five column volumes (CV) of running buffer (50 mM Tris/HCl, pH 7.4, 300 mM NaCl, 20 mM Imidazole), the fifth wash fraction showed an increased imidazole concentration (40 mM). The protein was eluted with five CV running buffer containing 250 mM imidazole. The eluted fractions were pooled and the buffer was exchanged with Tris‐buffer using a sepharose column (HiTrap Desalting, 5 mL, GE Healthcare). All elution fractions were pooled, the concentration determined with a BCA assay, and the proteins stored in Tris‐Buffer with 2 mM DTT at −80°C.

### 2.7 In‐vitro protein‐based cleavage assays

To build a substrate for in‐vitro protein‐based cleavage experiments, Human Ubiquitin‐conjugating Enzyme E2 L3 was *N‐*terminally linked to a fusion protein containing an *N‐*terminal His tag, a GSG linker, and a VDVAD recognition site for Caspase‐2.^73^ This resulted in the substrate sequence 6H‐GSG‐VDVAD‐X‐E2 where X can be any of the 20 canonical amino acids in order to be able to test the influence of the P1' site on cleavage relative to P1' Gly in both, the mutants and the unmutated protease.

A crucial goal in the design of the Caspase‐2 mutant was to minimize the risk of off‐target cleavage, thus to maintain the specificity of the S1 to S5 binding pockets at a level comparable to the unmutated protein. In order to be able to assess changes in binding specificity of the S1 to S5 pockets upon mutation, the influence of the mutations on binding were also assessed toward a variety of substrates with alternative recognition sequences and compared to the unmutated Casp‐2. Three alternative sequences were used which were *N‐*terminally linked to E2: (a) the sequence 6H‐GSG‐DEVD‐G‐E2. DEVD‐G is the preferred recognition sequence and site of cleavage of Caspase‐3. Since Caspase‐3 lacks an S5 subpocket,^34^ this recognition sequence has no dedicated P5 site, the Caspase‐2 S5 pocket binding to the Gly of the linker; (b) the sequence 6H‐GSG‐DETD‐R‐E2. DETDR is a pattern prominent within Caspase‐2 (residues 323‐327) that has an Arg residue at the P1' site. A more promiscuous binding site would bring the risk of possible autocleavage of the protein, rendering the protein inactive; (c) the sequence 6H‐GSG‐VDQQE‐G‐E2. VDQQE constitutes the C‐terminus of the large subunit in the unmutated Casp2 and is also the site of activation by autocatalytic cleavage within the protein. To quantify any change in cleavage specificity the time until 50% cleavage was measured in comparison to the time needed to cleave 50% of the 6H‐GSG‐VDVAD‐G‐E2 sequence.

To assess if the inserted mutations affect the stability of the mutants compared to the unmutated Casp2, the influences of higher incubation temperatures (37 and 50°C) and of chaotropic agents (tween 0.1%, urea 4 M, guanidinium chloride [GuHCl] 1 M, imidazole 0.5 M) on the protein mutants and the unmutated Casp2 were investigated.

All experiments were executed in an appropriate Caspase‐2 assay buffer^75^ at 25°C. The purified caspase‐2 mutants (1 mg/mL) were mixed with the substrate with an enzyme to substrate mass ratio of 1:100 for fast‐proceeding reactions or 1:10 for slow‐proceeding reactions (Asp, Glu, Ile, Val, and Pro) at room temperature in Caspase‐2 assay buffer (10 mM PIPES pH 7.2, 100 mM NaCl, 10% sucrose, 0.1% CHAPS, 1 mM EDTA, 10 mM DTT). Several samples were taken at timed intervals and the reaction was quenched immediately by addition of MagicMix SDS‐PAGE buffer (3.5 M Urea, 85 mM Tris HCL pH 6.8, 10% (v/v) glycerol, 2% (w/v) SDS, 0.01% (w/v) bromophenol blue, 0.7% (v/v) *β*‐mercaptoethanol). SDS‐PAGE assays were used to separate cleaved from uncleaved substrate. In order to assess the influence of P1' on cleavage relative to P1' Gly, the intensities of the bands resulting from the cleaved protein were quantified using the GE Healthcare ImageQuant TL 1D software (v. 8.1). To standardize the process only those samples showing about 50% substrate cleavage were used for the quantification of the cleavage. Examplary gels after different times of cleavage are shown in Figure S1. All experiments were executed in triplicates. Cleavage was defined as the time needed to cleave 50% of the substrate with amino acid X on the P1' site, normalized by the time needed to cleave 50% of a substrate with Gly on the P1' site. Casp2 (0.01 mg/mL) cleaved 50% of the substrate VDVAD‐G‐E2 (1 mg/mL) at 25°C in Caspase‐2 assay buffer within 1 min. These conditions were defined as the standard activity to which all other reactions were compared. This normalization was needed to make the results independent from protein purity and activity, which may vary due to expression and storage conditions.

### 2.8 In‐vitro peptide‐based cleavage assays

In‐vitro peptide‐based cleavage assays were used to determine Michaelis‐Menten parameters. These assays were executed for a limited set of P1' amino acids and utilized the FRET effect. The substrates were obtained from Bachem AG (Weil am Rhein, Germany) and were of the general structure of Abz‐VDVAD‐XA‐Dap(Dnp), where A, G, I, L, P, T, and V were substituted for X (the P1' position). 2‐Aminobenzoyl (Abz) was used as fluorophore and 2,4‐Dinitrophenyl (Dnp) was used as quencher. Diamino‐propionic acid (Dap) is used as a linker between the peptide and the quencher. A complete list of substrates can be found in Table S2. All substrates were dissolved in 10 mM HEPES, pH 7.5 to a concentration of 750 μM. The buffer for the assay was 50 mM HEPES, 150 mM NaCl, pH 7.2. The assay was calibrated by incubating varying amounts of substrate (20, 6.9, 2.4, 0.8, 0.3, and 0.1 μM) with 72 μM Casp2 in phosphate buffered saline (PBS). Each mixture was incubated at room temperature for up to 24 hours. Hundred percent conversion was assumed. Fluorescence was measured in black 96 well plates on a Tecan Infinite M200 Pro plate reader. Excitation wavelength was 320 nm, emission wavelength 420 nm. Michaelis‐Menten kinetics were measured by varying substrate concentrations (200, 100, 50, 20, and 10 μM) at a constant enzyme concentration of 1 μM. The initial slope was measured by measuring the fluorescence for 3 to 15 minutes (or 3‐20 hours for proline as P1', due to the slow kinetics) and calculating the slope of the initial measurement in μM product generated per second. Fluorescence was measured in black 96 well plates on a Tecan Infinite M200 Pro plate reader. Excitation wavelength was 320 nm, emission wavelength 420 nm. In the FRET assay all substrates, except for proline as P1' showed excellent linearity for at least a few minutes. Evaluation of the data was performed by fitting the data in the TableCurve 2D v5 software to a Michaelis‐Menten kinetic:

(3) $$v=\frac{V_{\max}\left[S\right]}{K_{M}+\left[S\right]}$$

with *v* being the initial slope, *V*_max_ is the maximum rate, *K*_*M*_ is the Michaelis constant, and [*S*] is the substrate concentration. The parameters *V*_max_ and *K*_*M*_ were fitted. *k*_cat_ was calculated by dividing *V*_max_ by the enzyme concentration [*E*].

## 3 RESULTS

### 3.1 Statistical analysis

**FIGURE 4:**
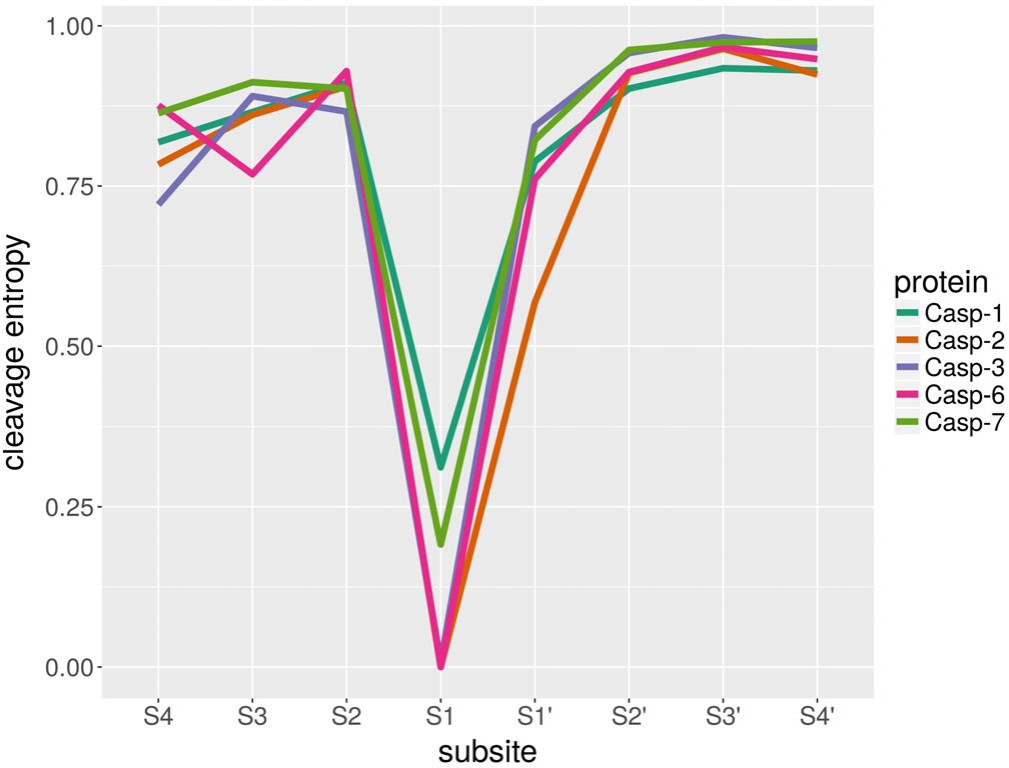
Pattern of cleavage entropies of subsites S4 to S4' for five different members of the human caspase family. Caspase‐2 S1' site shows the highest specificity while the same site in Caspase‐3 shows the highest promiscuity within all five members of this protease family. Also, the S1 site in Caspase‐1 and Caspase‐7 is less promiscuous with a cleavage entropy >0.0 and being less specific toward Asp than the other three family members [Color figure can be viewed at wileyonlinelibrary.com]

Analysis of cleavage data indicates that the S1' subsite of Caspase‐2, which favors small, unbranched or polar amino acids like Gly, Ser, and Ala over other amino acids (see Table 1) is the second‐most specific subsite after S1, which is exclusively binding to Asp. Evaluation of cleavage entropies of caspase family members Caspase‐1, Caspase‐3, Caspase‐6, and Caspase‐7 reveals the differences within this set of related proteases (Figure 4). In Caspase‐2, the S1' subsite shows a relatively low cleavage entropy compared to the subsites S2', S3', and S4' (Table S1). The S1' binding sites of the other caspases show enhanced promiscuity compared to Caspase‐2, while S2', S3', and S4' sites remain unchanged in terms of cleavage entropies. These findings render all of these proteins interesting templates for the optimization of the Caspase‐2 S1' binding site. Caspase‐3 shows the highest cleavage entropy at the S1' site compared to its family members, while having an overall lower cleavage entropy pattern at the sites S4 to S2. The Caspase‐3 S1 site, on the other hand, shows the same specificity as the S1 site in Caspase‐2 (Table S1), as it selectively detects Asp residues. For this reason, we chose Caspase‐3 as a template to further optimize the S1' pocket promiscuity of Caspase‐2.

**TABLE 1:** Amino acid distribution in Caspase‐2 and Caspase‐3 substrates

P1'	Caspase‐2	Caspase‐3
Gly	0.488	0.264
Ser	0.174	0.139
Ala	0.136	0.108
Met	0.059	0.033
Cys	0.030	0.045
Phe	0.023	0.047
Trp	0.018	0.020
Val	0.018	0.018
Tyr	0.017	0.060
Asn	0.012	0.081
His	0.009	0.031
Gln	0.005	0.009
Thr	0.004	0.032
Leu	0.004	0.019
Glu	0.003	0.011
Arg	—	0.018
Lys	—	0.008
Asp	—	0.027
Ile	—	0.020
Pro	—	0.010

*Note*: Probability distribution of amino acids occurence in the P1' sites among substrates cleavable by Caspase‐2 and Caspase‐3. These values were normalized by the natural occurrence of the amino acids in humans (as found in the UniProt Knowledgebase^44^) and to sum up to 100%. For both proteases, small, polar and unbranched amino acids are preferred substrates over branched, hydrophobic, or charged amino acids. Overall, Caspase‐3 has a more flat probability distribution of amino acids. Frequencies without numbers lack any observation in the statistical data.

### 3.2 Sequence and structural analysis

Comparing the aligned Caspase‐2 and Caspase‐3 sequences gave a total sequence identity of only 26.2%. However, the active sites of these family members are more conserved: comparing the sequences that make up the active site only in both proteins gave a sequence identity of 50.0% (Figure S2), which further supports our choice of using Caspase‐3 as a promising candidate to serve as a template for enhancing the Caspase‐2 P1' site promiscuity.

**FIGURE 5:**
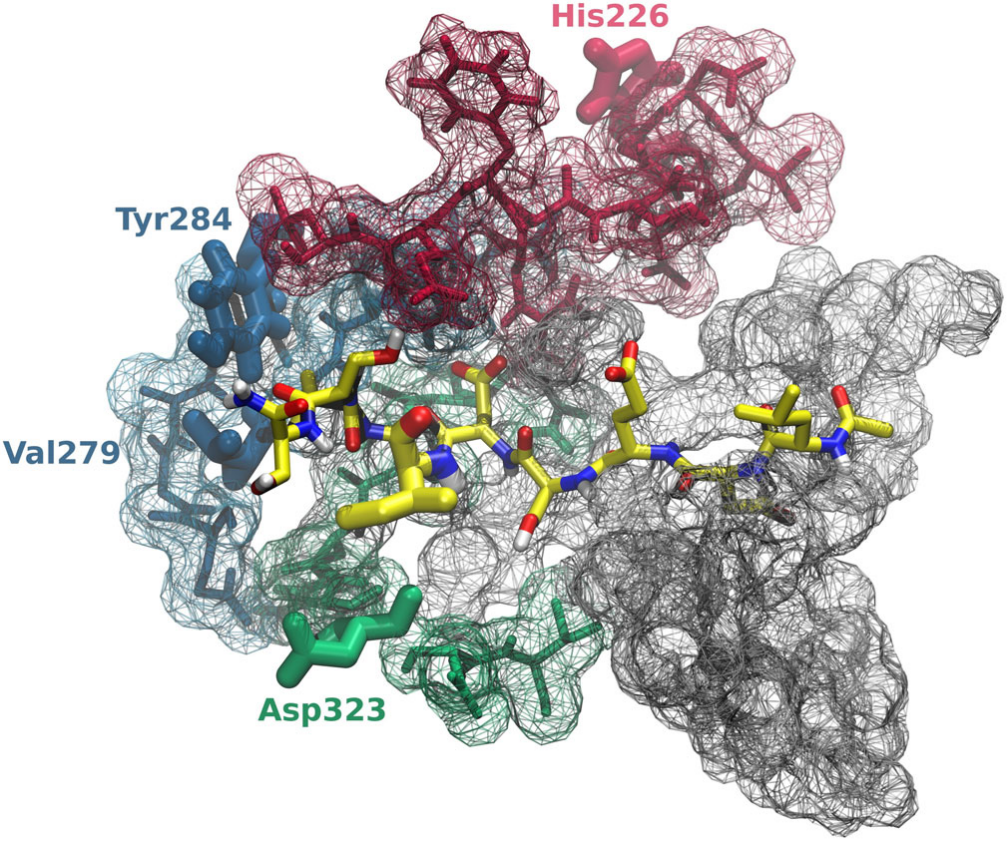
Visualization of Caspase‐2 active site. The entire active site is shown using a grid representation. Those strands interacting with the substrate P1' to P4' sites are shown using a stick representation (different strands were assigned the individual colours green, blue and red). The four selected candidate mutants and the P1' site (shown in green) are represented as bold sticks. The P1' site is shown in bold stick representation, while the rest of the substrate is shown in thin stick representation (yellow carbon atoms) [Color figure can be viewed at wileyonlinelibrary.com]

Structural analysis of Caspase‐2 shows that unlike other substrate binding sites, there is no explicit cavity to host the P1' side chain. The fact that the S1' binding site only shows tolerance toward a limited set of small amino acids is potentially related to the lack of a real S1' binding site. This makes the problem at hand, that is, optimizing the P1' promiscuity, rather complex, since no amino acids directly linked to the S1' pocket can be identified and considered as potential targets for mutation and optimization. Additionally, any mutation that has an impact on P1' binding, might affect the binding at the other substrate binding sites as well. Based on distances, all amino acids that differ between Caspase‐2 and Caspase‐3 active site strands were evaluated. Only those amino acids were marked as potential candidates for mutation, that are located on strands that (after visual analysis) interact directly only with the substrate P1' to P4' sites, but not with P1 to P4. Using this procedure, only four amino acid mutations were identified as potential candidates: His226Ala, Val279Glu, Tyr284Phe, and Asp323Thr (Figure 5, sequence numbers according to acc. number P42575). The latter three amino acid mutations are simply in close proximity to the substrate P1' to P4' sites and directly interact with them. The distances between the closest atoms of the respective residues and P1' Ile of the equilibrated structure are 4.2 Å(Asp323), 5.9 Å(Val279), and 6.4 Å(Tyr284). The mutation Asp323Thr eliminates a negative charge in close proximity of the P1' binding site, while the mutation Val279Glu introduces a negative charge. Both might energetically affect the conformational freedom of the substrate. The mutation His226Ala might act in a more indirect way. Even though His226 is not directly interacting with the substrate P1' to P4' sites, its hosting strand is. His226 is believed to stabilize the hosting strand as it was seen in our MD simulations to act as both, hydrogen‐bond donor and acceptor with Ser206 (75.7% as donor of the simulation time/8.8% as acceptor) and Cys244 (40.9% as donor of the simulation time/0.4% as acceptor). Mutating His226 into an amino acid that is not capable of forming hydrogen bonds (such as Ala) may render the entire hosting strand more flexible. Since it was shown before that there is a relationship between active site flexibility and specificity in caspases,^76^ His226 was considered a potential candidate for mutation.

### 3.3 Free energy calculations

#### 3.3.1 Calculations in the tetrahedral intermediate state

The impact of the four selected candidate mutations on the tetrahedral intermediate state was determined using free energy calculations. The sequence for the P5 to P4' model substrate was chosen to be Leu‐Asp‐Glu‐Ser‐Asp‐Ile‐Val‐Ser‐Ser. The sequence from P5 to P1 was already bound to the protein in the used crystal structure. The sequence from P1' to P4', on the other hand, was missing in the crystal structure, and was chosen such that the P1' site bears one of the least preferred residues according to the statistical data (Table 1). Val (P2') and Ser (P3'‐P4') are among the most preferred residues, according to the statistical data (data not shown). The P2' to P4' sites show high cleavage entropies (Figure 4), thus the exact choice of amino acids here seems not to be of major importance.

**FIGURE 6:**
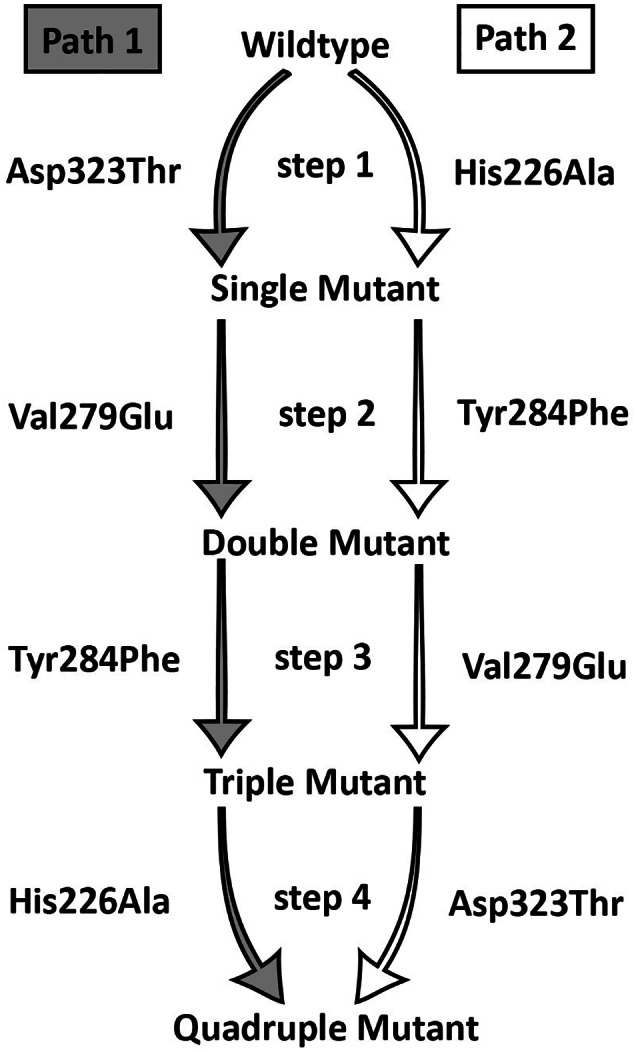
Thermodynamic cycle for the free energy calculations of the four selected candidate mutations. Two alternative paths were chosen to mutate the unmutated protein into the full quadruple mutant, where the free energy change upon mutation of the individual amino acids were calculated in the exact reverse direction

To quantify the individual contributions of each of the four candidate mutations, as well as their potential dependencies on substrate binding, free‐energy calculations were performed according to a thermodynamic cycle. This cycle was constructed such, that the full path of mutating the unmutated protein into the quadruple mutant (four mutations) was calculated twice, where the sequential mutations were calculated in exactly reverse order. By doing so, any mutation in path 1 would follow prior mutations that were not seen yet in path 2, and vice versa (Figure 6). This procedure has another advantage, which is, that cycle closure can be assessed. Although, in the case of possible dependencies between the individual mutations one could not expect to get exactly the same free energy values for the same mutation in both paths, the total sum over the four sequential free‐energy calculations should give the same result for both paths. If not, this indicates potential issues related to insufficient sampling and convergence. All of these mutations were introduced with the substrate in the tetrahedral intermediate state.

Independent of the path, two of the four selected candidate mutations (Asp323Thr and His226Ala) involve a negative change in binding free energies, thus are predicted to have a favorable effect on the stability of the complex (Table 2). The mutation Val279Glu shows the strongest effect. The free‐energy changes of 11.7 kJ/mol (path 1) and 9.7 kJ/mol (path 2) indicate a very negative impact on the stability. The free‐energy change for the mutation Tyr284Phe is positive, but closer to zero and for path 2 even within the error, so this mutation might even have no or only little effect on the tetrahedral intermediate. The mutation His226Ala shows the strongest dependence on other mutations, with an absolute difference of 4.6 − 1.9 = 2.7 kJ/mol between both paths. Importantly, the total sums of free energy changes of mutating the unmutated Casp2 into the quadruple mutant in both paths add up to a very small (less than kT) absolute error of 2.7 − 2.1 = 0.6 kJ/mol. The corrections related to charge‐changing perturbations affect these values (differences between ΔΔ*G*_raw_ and ΔΔ*G*_TI_) only to a limited extent.

**TABLE 2:** Results from free energy calculations with the protein‐substrate complex in the tetrahedral intermediate (TI) state

Path	Step	Mutation	ΔΔ*G*_*TI*_	(ΔΔ*G*_raw_)
1	1	Asp323Thr	−7.5 ± 0.8	(−7.2)
	2	Val279Glu	11.7 ± 4.0	(9.9)
	3	Tyr284Phe	2.5 ± 1.7	
	4	His226Ala	−4.6 ± 2.0	
		TOTAL	2.1 ± 4.8	(0.7)
2	1	His226Ala	−1.9 ± 0.6	
	2	Tyr284Phe	1.8 ± 3.0	
	3	Val279Glu	9.7 ± 2.6	(6.5)
	4	Asp323Thr	−6.9 ± 4.3	(−4.2)
		TOTAL	2.7 ± 5.9	(2.2)

*Note*: The values of all four steps of both paths are shown. ΔΔ*G*_*TI*_ denotes the (corrected) binding free energies, while ΔΔ*G*_raw_ denotes the uncorrected binding free energies for the mutations that involve net‐charge changes. All values are reported in kJ/mol. Error estimates indicate standard deviations over three independent simulations and over the two active sites in the dimeric structure.

#### 3.3.2 Calculations for the noncovalently bound state

Two of the four selected candidate mutations (Asp323Thr and His226Ala) were predicted to have a favorable effect on the stability of the tetrahedral intermediate. However, these free energies correspond to the difference of the covalently bound substrate complex vs the unbound state. As such, they represent the sum of the binding process and the first, rate determining, step in the catalytic mechanism. From the difference between ΔΔ*G*_TI_ and ΔΔ*G*_bind_, also the difference for the first catalytic step, ΔΔ*G* ^‡^ was estimated (assuming independence of the values for single mutations). It was found that ΔΔ*G* ^‡^ was small and within the statistical uncertainty, mostly because the uncertainties for the simulations of the noncovalently bound state are higher (compare standard deviations for steps 1 and 4 which are shown in Tables 2 and 3).This was expected, due to the higher conformational freedom of the substrate, which potentially also allows for the inclusion of nonproductive binding modes in ΔΔ*G*_bind_. To check for this, we monitored the distance between the carbonyl carbon and Cys220 in these simulations and indeed found distances larger than 0.4 nm for 67% of the time. For this reason, we judge the values of ΔΔ*G*_*TI*_ to be more representative for the catalytic process.

**TABLE 3:** Results from free energy calculations with the protein‐substrate complex not in the tetrahedral intermediate state

Path	Step	Mutation	ΔΔ*G*_bind_	(ΔΔ*G*_raw_)	ΔΔ*G* ^‡^
1	1	Asp323Thr	−11.2 ± 3.4	(−11.0)	3.7 ± 2.3
	2	His226Ala	−4.8 ± 3.2		0.2 ± 2.7
		Asp323Thr/His226Ala	−16.0 ± 4.7	(−15.8)	
2	1	His226Ala	−2.4 ± 2.1		0.5 ± 1.6
	2	Asp323Thr	−13.5 ± 4.4	(−13.3)	6.6 ± 4.1
		Asp323Thr/His226Ala	−15.9 ± 5.3	(−15.7)	

*Note*: The results of steps 1 and 4 of both paths are shown. ΔΔ*G*_bind_ denotes the (corrected) binding free energies, while ΔΔ*G*_raw_ denotes the uncorrected binding free energies for the mutation that involves net‐charge changes. ΔΔ*G* ^‡^ was calculated as the difference between ΔΔ*G*_*TI*_ (see Table 2) and ΔΔ*G*_bind_. All values are reported in kJ/mol. Error estimates indicate standard deviations over three independent simulations and over the two active sites in the dimeric structure.

#### 3.3.3 Calculations in the substrate P5 to P1 sites

The main goal of the underlying work was to modify the active site such that the S1' site becomes more promiscuous toward different amino acids. However, to ensure that the protease is suitable for targeted *N*‐terminal cleavage, the selectivity of the S1 to S5 subsites has to be kept as high as possible. Changes in the selectivities of these pockets were assessed by mutating the P5 to P1 sites of the bound substrate in the tetrahedral intermediate state into Ala twice: in the double mutant and in the unmutated protease. Table 4 reveals that no significant differences in free energies between the two proteins could be found. According to these calculations, the promiscuity of the S5 to S1 binding pockets was not affected by the mutations.

**TABLE 4:** Results from free energy calculations in the substrate P5 to P1 sites in the protein‐substrate complex in the tetrahedral intermediate (TI) state

	Δ*G*_*x* → Ala_ (wt)	Δ*G*_*x* → Ala_ (D323T/H226A)	ΔΔ*G*
P5	−2.1 ± 1.3	−2.5 ± 0.5	−0.4 ± 1.4
P4	422.2 ± 1.2	421.9 ± 3.8	−0.3 ± 4.0
P3	400.9 ± 4.5	401.8 ± 2.9	0.9 ± 5.4
P2	45.9 ± 1.1	47.2 ± 1.8	1.3 ± 2.1
P1	476.5 ± 2.4	477.8 ± 4.3	1.3 ± 4.9

*Note*: No significant differences could be found, revealing that substrate promiscuity of the S5 to S1 was not affected by the mutations. All values are reported in kJ/mol. Error estimates indicate standard deviations over three independent simulations and over the two active sites in the dimeric structure.

### 3.4 In‐vitro protein‐based cleavage assays

**FIGURE 7:**
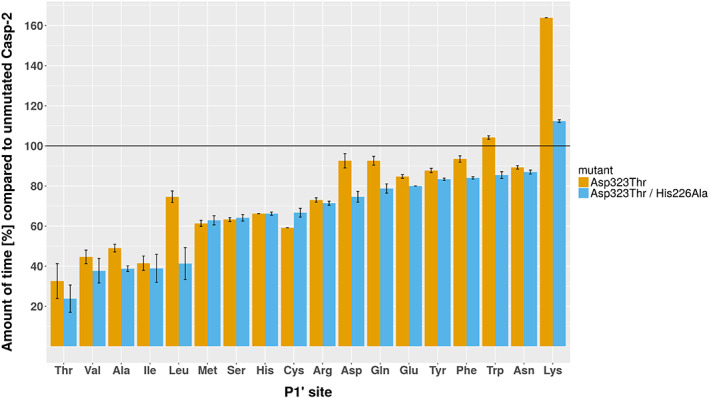
Influence of P1' on cleavage relative to P1' Gly as determined from protein‐based cleavage assays. Reported is the relative time to cleave 50% of the substrate. Both mutants show a significant decrease in cleavage time toward branched amino acids. The horizontal bar at 100% marks the unmutated Casp‐2 as a reference. Cleavage of P1' Pro was too slow in protein‐based cleavage experiments to report meaningful values. Underlying values are reported in Table 5 [Color figure can be viewed at wileyonlinelibrary.com]

**FIGURE 8:**
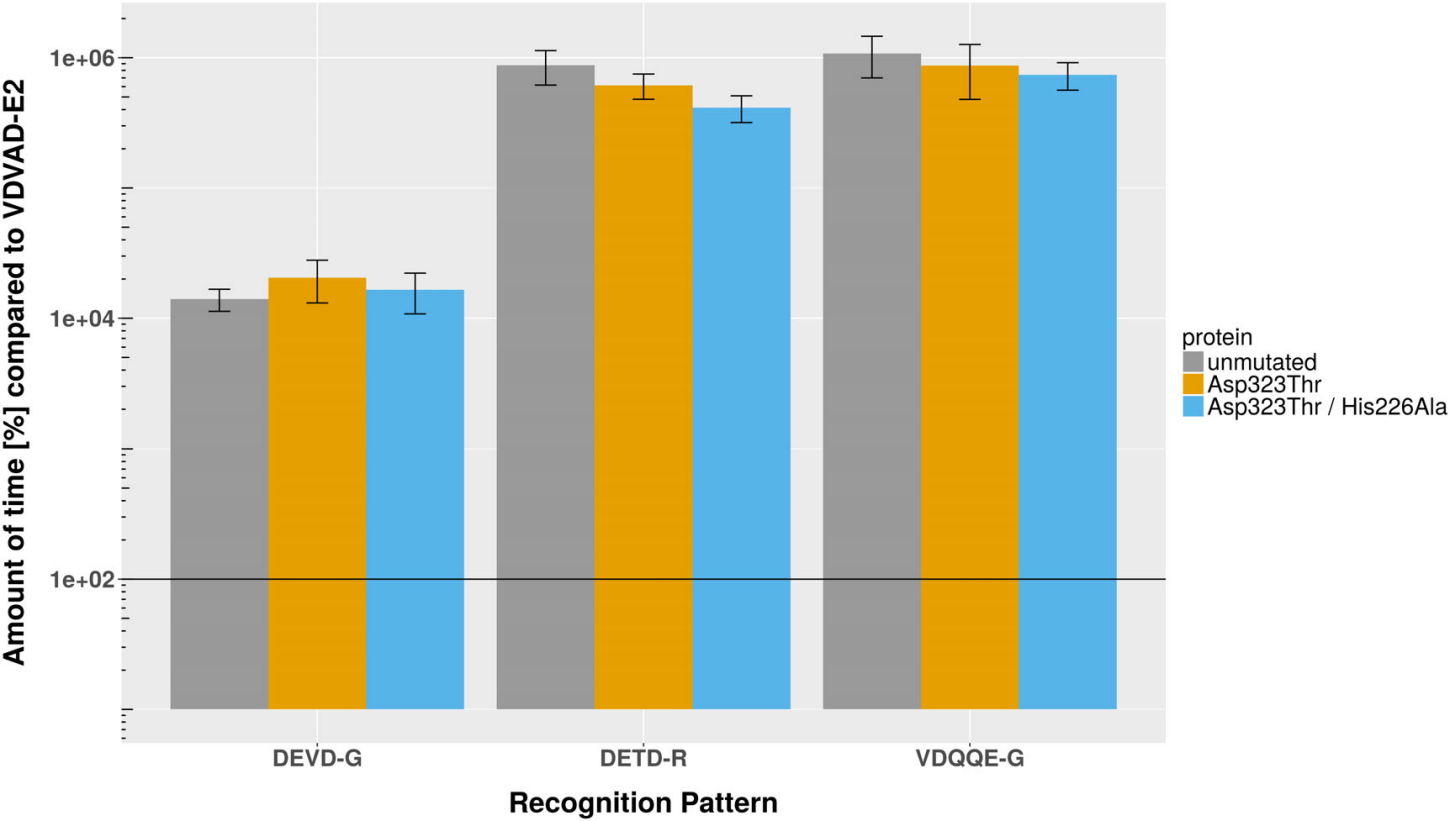
Experimentally determined specificities from protein‐based cleavage assays using a set of alternative recognition sequences. The time needed to cleave 50% of the substrate increases by two orders of magnitude toward DEVD‐G and by four orders of magnitude toward the DETD‐R and VDQQE‐G sequences compared to the native recognition site (VDVAD‐G‐E2). There are no significant differences between the single mutant, the double mutant and the unmutated Casp‐2. Note the logarithmic scale on the *y*‐axis [Color figure can be viewed at wileyonlinelibrary.com]

To evaluate the influence of the two mutations that were predicted to favor Ile at Position P1' of the substrate according to the free energy calculations, two mutants were generated and the influence of P1' on cleavage relative to P1' Gly was assessed: a single mutant with the mutation Asp323Thr and a double mutant, which was based on the single mutant with the additional mutation His226Ala. To avoid differences in protein purification and concentration, cleavage was defined as the time needed to cleave 50% of the product relative to this value for the substrate with Gly at P1'. Cleavage experiments showed enhanced promiscuity toward the P1' site, with both mutants roughly halfing the cleavage times compared to the unmutated protease toward threonine, valine, isoleucine and alanine at the P1' site, while the double mutant also exhibited such behavior toward leucine at the P1' site. In contrast, the time for cleavage increased for lysine at the P1' site, to roughly 200% in the case of the single mutant and only marginally (to ~125%) for the double mutant. The tolerance with respect to all other remaining amino acids as P1' binding partners did not change significantly (Figure 7 and Table 5). The S1 to S5 subsites of the single mutant showed no significant changes in specificity toward the recognition sequences 6H‐GSG‐DEVD‐G‐E2, 6H‐GSG‐DETD‐R‐E2, and 6H‐GSG‐VDQQE‐G‐E2 compared to the unmutated Casp2 (Figure 8). For these recognition sites, the cleavage times were increased by 2 to 4 orders of magnitude compared to the optimal recognition site 6H‐GSG‐VDVAD‐G‐E2.

**TABLE 5:** Influence of P1' on cleavage relative to P1' Gly as determined from protein‐based cleavage assays

P1'	Unmutated	D323T	D323T/H226A
Thr	180 ± 12.0	58.0 ± 16.4	42.4 ± 6.8
Val	631.3 ± 98.0	281.6 ± 40.9	238.0 ± 54.7
Ala	44.7 ± 11.8	22.0 ± 2.0	17.3 ± 0.9
Ile	1269.3 ± 343.5	529.3 ± 75.1	494.4 ± 134.8
Leu	400.0 ± 117.6	296.9 ± 64.9	165.1 ± 54.3
Met	35.7 ± 2.3	22.0 ± 2.0	22.5 ± 3.2
Ser	12.5 ± 1.7	7.9 ± 0.5	8.0 ± 0.8
His	52.4 ± 10.9	40.0 ± 4.2	34.7 ± 1.8
Cys	5.6 ± 0.7	3.3 ± 0.4	3.8 ± 0.5
Arg	20.2 ± 2.8	14.8 ± 1.2	14.5 ± 1.1
Asp	713.6 ± 41.7	699.0 ± 226.7	562.0 ± 113.0
Gln	209.1 ± 71.6	193.9 ± 38.5	165.0 ± 29.9
Glu	3010.3 ± 805.8	2548.7 ± 170.3	2400.0 ± 145.1
Tyr	37.8 ± 1.8	33.0 ± 3.2	31.5 ± 2.0
Phe	20.6 ± 6.5	19.3 ± 2.9	17.3 ± 0.9
Trp	28.8 ± 1.0	30.0 ± 3.4	24.6 ± 3.5
Asn	22.7 ± 5.0	20.3 ± 1.6	19.7 ± 1.5
Lys	24.4 ± 7.1	40.0 ± 3.5	27.3 ± 2.2
Gly	1	1	1

*Note*: Reported is the time to cleave 50% of the substrate relative to Gly (approx. 1 min in the unmutated protein). Cleavage of P1' Pro was too slow in protein‐based cleavage experiments to report meaningful values.

The enzyme stability of the two mutants, as assessed by the means of elevated temperature and chaotropic agents, showed no altered behavior compared to the unmutated Casp2. 0.1% Tween had no significant impact on cleavage for neither of the compared protein variants, while 4 M urea, 1 M GuHCl, and 0.5 M imidazole exerted the same hindering effects on all variants (Figure S3). Also, cleavage experiments at elevated temperatures showed no significant differences within the timeframe of these experiments (Figure S4), suggesting that the protein stability is unperturbed by the mutations.

### 3.5 In‐vitro peptide‐based cleavage assays

**FIGURE 9:**
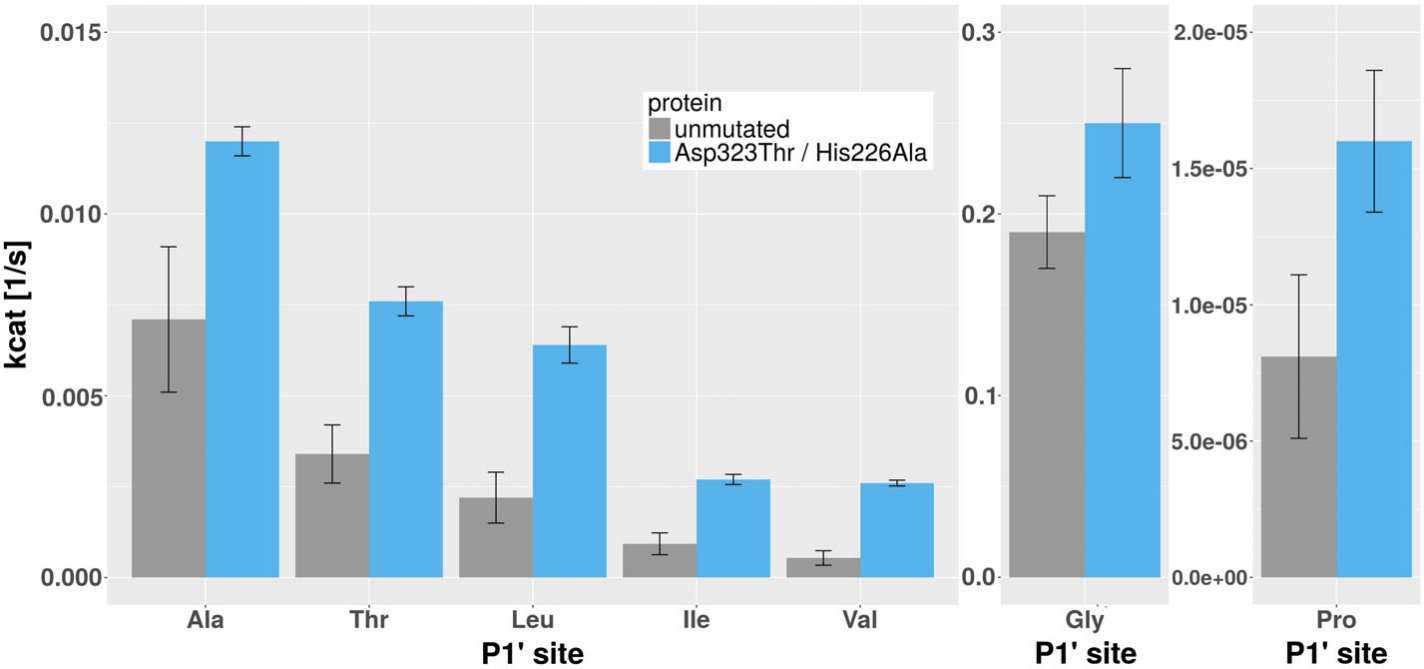
Experimentally determined *k*_cat_ values from peptide‐based cleavage assays. A set of branched apolar and polar amino acids as well as Ala, Gly, and Pro were tested at the substrate P1' site. The double mutant shows roughly doubled catalytic efficiency for all amino acids tested except Gly [Color figure can be viewed at wileyonlinelibrary.com]

A peptide‐based cleavage assay utilizing FRET for real time reaction monitoring was used to determine the Michaelis‐Menten kinetic parameters of the unmutated Casp2 and the double mutant. The parameters, shown in Table 6, reveal an elevated catalytic efficiency of the mutant relative to the unmutated protease, as seen from increased *k*_cat_/*K*_*M*_ values, regardless of the tested P1' amino acids. Due to fluorescent signal quenching at higher substrate concentrations, the confidence intervals for the *K*_*M*_ values are rather high. Additionally, the influence of the P1' amino acids on the catalytic efficiency was much more apparent in the turnover number *k*_cat_, where an experimental dynamic range of more than 30 000 was covered (from 8 × 10^−6^ s^−1^ to 0.3 s^−1^). In comparison, the dynamic range of the recorded *K*_*M*_ values was roughly 7. Because of the larger influence on enzymatic activity and the increased experimental accuracy, the comparison of the turnover number *k*_cat_ was found to be a more meaningful predictor of Casp‐2 activity (see Figure 9).

**TABLE 6:** Enzymatic parameters derived from Michaelis‐Menten kinetic FRET experiments for unmutated Casp2 and the double mutant carrying the mutations Asp323Thr and His226Ala

P1'	Enzyme	*K*_*M*_ (μM)	*k*_cat_ (s^−1^)	Relative *k*_cat_	*k*_cat_/*K*_*M*_ (M^−1^ s^−1^)
Ala	Unmutated	89 ± 11	7.1 ± 0.4 × 10^−3^		80
	D323T/H226A	57 ± 23	1.2 ± 0.2 × 10^−2^	172%	214
					
Gly	Unmutated	112 ± 37	1.9 ± 0.3 × 10^−1^		1720
	D323T/H226A	55 ± 13	2.5 ± 0.2 × 10^−1^	127%	4511
					
Ile	Unmutated	71 ± 25	9.3 ± 1.4 × 10^−4^		13
	D323T/H226A	67 ± 18	2.7 ± 0.3 × 10^−3^	286%	40
					
Leu	Unmutated	287 ± 96	2.2 ± 0.5 × 10^−3^		7.5
	D323T/H226A	193 ± 35	6.4 ± 0.7 × 10^−3^	295%	33
					
Pro	Unmutated	305 ± 146	8.1 ± 2.6 × 10^−6^		0.026
	D323T/H226A	43 ± 23	1.6 ± 0.3 × 10^−5^	201%	0.37
					
Thr	Unmutated	75 ± 20	3.4 ± 0.4 × 10^−3^		45
	D323T/H226A	82 ± 18	7.6 ± 0.8 × 10^−3^	225%	94
					
Val	Unmutated	64 ± 23	5.4 ± 0.8 × 10^−4^		8.5
	D323T/H226A	77 ± 14	2.6 ± 0.2 × 10^−3^	480%	34

*Note*: The values for *K*_*M*_ and *k*_cat_ include the 95% confidence interval of the model fit, based on three measurements at five different substrate concentrations (n = 15).

## 4 DISCUSSION

In this work, Caspase‐2 was modified in‐silico to allow for a more promiscuous binding of the S1' subsite. The approach we chose was to search for a suitable template protein, which bears the features we were trying to modify in Caspase‐2, but is also close enough to our protease of interest in terms of sequence‐ and structural similarity. We found a suitable protein in human Caspase‐3 (Caspase‐3). Comparison of statistical data^29^ between all human caspases revealed that the Caspase‐3 S1' subsite shows the highest promiscuity within this protein family. Simultaneously the active site structure between Caspase‐2 and Caspase‐3 is highly conserved. By structural analysis, we identified all residues which differ between Caspase‐2 and Caspase‐3 and are in close proximity to the S1' binding site. These candidate mutations were believed to have a possible positive impact on S1' (and S2'‐S4') subsite promiscuity while hardly affecting the specificity of the S1 to S5 subsites. The effects of these were further assessed by calculating the stability of the tetrahedral intermediate in terms of free energies for a model substrate that is not particularly well cleaved by unmutated Caspase‐2. That is why a substrate bearing an Ile residue in the P1' position was chosen. In fact, Ile does not show up as an accepted binder of the Caspase‐2 S1' pocket according to the statistical data, while it has a nonzero probability for Caspase‐3 (Table 1).

Free energies were calculated by employing alchemical perturbations of the candidate mutations along a *λ*‐parameter in the protein‐substrate complex and in the apo‐protein. Some of the modifications involved residues with different net charges, requiring special attention in the calculations of free energies, specifically the “raw” charging free energies. These “raw” charging free energies are known to be very sensitive toward the used simulation methods, but can be corrected *ex post*.^65^, ^66^ We did so by a set of computationally and analytically derived terms, here simply called correction terms. However, we note that most of the artifacts related to charge‐changing perturbations cancel out along the applied thermodynamic cycle. In other cases, for example, substrate perturbations involving charge changes, one cannot rely on error cancellation. The reason is that the individual legs of the thermodynamic cycle to calculate relative free energies of binding are represented by systems which might be more dissimilar, for example, a substrate bound to a solvent‐excluded cavity vs a substrate completely solvated in buffer. In such cases, applying the charging corrections has a significantly bigger effect, potentially even changing favorable into unfavorable free energies of binding (or vice versa).^43^

Two of the suggested mutations were computationally predicted to increase the affinity for Ile in P1'. We mainly focused on simulations for the tetrahedral intermediate state, which is expected to be close to the reactive transition state. Hence, the calculated free energies are associated to the combined effect of substrate binding and the first catalytic step. It should be noted that that the energetic change of bond formation is a crucial step toward the tetrahedral intermediate, which cannot be assessed in a satisfying way using classical MD simulation. However, assuming that the mutations do not affect the actual bond formation, but stabilize or destabilize the tetrahedral intermediate through nonbonded interactions, a relative free energy, ΔΔ*G*_*TI*_ can be estimated using Figure 3. In an attempt to relate the free energies more precisely to the measured kinetic parameters, the relative binding free energies for the noncovalently bound complexes were also computed. A larger spread in the six independent estimates was obtained, suggesting a larger variability in the binding poses. Indeed, a large portion of the sampled conformations were nonproductive binding modes in which the carbonyl carbon was too far away from the Cys S_*γ*_ atom. This hampers a direct comparison to the *K*_*M*_ values, as these are representative of the productive binding. Accordingly the values of ΔΔ*G* ^‡^ are rather noisy, and not comparable to *k*_cat_ directly. Therefore, we focus on the values for ΔΔ*G*_*TI*_ instead, in which the covalent bond ensures sampling of the relevant phase space.

Protein‐based cleavage assays (Figure 7 and Table 5) revealed that both proposed mutants indeed cleave faster if the substrate bears a branched or hydrophobic residue at the P1' site, compared to the unmutated protease. While the trends were captured correctly in the free‐energy calculations, a quantitative comparison is difficult. A ΔΔ*G*_*TI*_ of 7 kJ/mol for the mutation Asp323Thr corresponds to a factor of roughly 16 in the *k*_cat_/*K*_*M*_. However, we only observed a factor of roughly 2.5 for Ile in the reaction efficiency in the protein system (Table 5) and of 3 in the *k*_cat_/*K*_*M*_ of the FRET assay (Table 6). Note, however, that the tetrahedral intermediate is not the kinetically relevant transition state, such that a direct comparison to kinetic data remains difficult. Furthermore, protein purity and activity varied due to protein expression and storage conditions, leading to uncertainty in the experimental data. To make the mutants comparable with the unmutated protein, we normalized all data from the protein‐based cleavage assays by Gly. However, these complications make it impossible to detect small changes in reactivities of the mutants.

For a more detailed kinetic analysis, we measured *k*_cat_ and *K*_*M*_ values for five amino acids that showed the highest decrease in cleavage time, as well as Gly and Pro in the double mutant. First, these experiments confirmed that the two mutations have a net positive effect on enzymatic activity. The intrinsic reactivity seems comparable between the unmutated protease and the double mutant, as exemplified in the turnover number *k*_cat_ for Gly in the P1' position (0.19 ± 0.03 s^−1^ for the unmutated protease vs 0.25 ± 0.02 s^−1^ for the double mutant). The activity for branched substrates on the other hand is greatly increased, with turnover numbers 2.2‐ to 4.8‐fold higher for the mutant. Interestingly, the mutation also seems to benefit the very slow cleavage of substrates with Pro at P1', where the turnover number was increased twofold and *k*_cat_/*K*_*M*_ increased by a factor 14.

Of the four probed mutations, the mutations Val279Glu and Tyr284Phe show an unfavorable change in free energies, where Val279Glu is prominently unfavorable compared with the other three mutations. The total free energy change of the quadruple mutant sums up to a value that is not significantly different to zero (Table 2). If one assumes that the four probed mutations are the main factor for the decreased specificity of the S1' binding pocket of Caspase‐3, one would not predict highly favorable binding of Ile to the Caspase‐3 S1' pocket. Indeed, the statistical data proves that Ile is a binder of the Caspase‐3 S1' pocket, but only a very poor one (Table 1). By selecting only two of the four mutations, we could increase the reactivity for Ile in Caspase‐2.

The normalized cleavage data was also used to recalculate “experimental” cleavage entropies for the mutants from the experimental data. Here, the experimental data was treated in the same way as the statistical data. While it is not meaningful to translate cleavage times to a probability measure (as needed for the calculation of cleavage entropies, see Equation (1)), the experimental cleavage profile of a protease is believed to be the same as the (hypothetical) statistical cleavage profile. In this respect, the calculation of experimental cleavage entropies (after translation of the data to probabilites) is a relevant measure. Experimental cleavage entropies were calculated for the unmutated Caspase‐2, the single as well as the double mutant. For the unmutated Casp‐2, the calculated experimental cleavage entropy (0.50 ± 0.04) was remarkably close to the statistical cleavage entropy (0.56, see Table S1). The experimental cleavage entropies for the two proposed mutants (single mutant: 0.56 ± 0.02, double mutant: 0.58 ± 0.02) are far from reaching a promiscuity level that is comparable to the cleavage entropy of the Caspase‐3 S1'‐pocket (0.84, see Table S1). However, while cleavage entropies are a measure of overall binding promiscuity, both mutations were selected to render the mutants S1' site a better binder of Ile. Thus, is does not come with a surprise that the experimental cleavage entropies reveal only a moderate increase in overall binding promiscuity of the S1' pocket.

In the current work, a suitable template protein could be found to guide modifications for the protein of interest. In cases where this is not possible, the search space for 10 amino acids involved in substrate binding involves 10^19^ possible mutations. Testing all of these is impossible with current methods and resources. Furthermore, computationally redesigned proteins often turn out to be less stable under experimental conditions, which might be related to the problem that even minimal changes in the amino‐acid sequence can often lead to unexpected changes in loop conformations, unfolding, or aggregation.^77^ However, in recent years, many methods and tools were described that enable the reliable prediction of a large variety of potential mutations. Fast methods were developed that do not rely on computionally demanding free‐energy calculations but predict the effect of mutations from easier‐to‐calculate parameters like amino‐acid occlusion from solvent, pairwise potentials and inter‐molecular energies.^78^, ^79^ Another succesfull approach was to combine computational algorithms with methods of experimental screening of protein‐libraries to design an ubiquitin‐ligase for binding to an unnatural interface.^80^ New methods to extend sampling while saving on computational costs like the weighted‐ensemble strategy^81^, ^82^ were developed and used effectively in the redesign of a protein conformational switch.^83^ Software suites like ROSETTA and ORBIT were succesfully used for de novo protein design.^83^, ^84^, ^85^, ^86^, ^87^, ^88^, ^89^ ROSETTA makes use of an extended energy function including reference energy terms for discriminating between protein mutants. It was generalized to work in many different contexts and is widely used to efficiently discriminate between mutants.^90^

It was shown that free‐energy differences for a large number of different substrates can be calculated from single simulations of unphysical reference states in combination with a third‐power fitting approach to capture the effects of molecular dipoles or charged states.^91^, ^92^ To screen for more mutants, these methods are currently employed by the authors to perform in silico saturation mutagenesis of Caspase‐2.

These advances and successes indicate the applicability of modern protein design methods. Altough enzymes designed with computational aid do usually not meet the efficiencies of natural enzymes, they can often be further improved by directed evolution.^93^ It can be summarized that protein engineering has become a robust and reliable field, also when de‐novo methods have to be applied or proteins have to be reingeneered without templates.

## 5 CONCLUSION

The restoration of the native *N*‐terminus after protein purification is of great importance in pharmaceutical industry. However, it remains a challenging task—due to the manifold characteristics of proteins. Protocols for hydrolysis, that is, cleavage at a specific position in a protein sequence to retrieve a desired *N*‐terminus, usually have to be optimized for every protein individually, which is a high‐cost factor for industrial production of recombinant proteins. While the target sequence for cleavage from the site of hydrolysis toward the *N‐*terminus can be chosen freely and optimized for the protease which is chosen for tag removal, this is not true for the target sequence in the *C‐*terminal direction from the site of hydrolysis, since this sequence constitutes the *N‐*terminus of the fusion protein. A protease used for universal tag‐removal thus requires highly specific binding pockets in *N‐*terminal direction from the site of hydrolysis and rather promiscuous binding behavior in *C‐*terminal direction from the site of hydrolysis.

In this work, human Caspase‐2 was engineered to yield a more promiscuous S1' subsite. A template protein, human Caspase‐3, which possesses a less specific S1' subsite, was used to predict possible mutations of the Caspase‐2 active site. Free‐energies of binding were calculated with a substrate model that features an Ile residue at the P1' binding site. The change of binding affinity of this substrate was assessed for four different candidate amino acid mutations, which were selected based on the sequence comparison between Caspase‐2 and Caspase‐3 active sites, followed by a structural analysis of the Caspase‐2 active site. The latter step in this workflow was chosen to filter active site amino acids by their subpockets in order to preserve the desired specificity of these sites.

Two suggested mutants based on the free‐energy calculations were tested in‐vitro. Changes in P1' substrate promiscuity were assessed by measuring the influence of P1' on cleavage relative to P1' Gly and by measuring Michaelis Menten parameters for a limited set of amino acids. Both mutants showed a significant change in activity toward substrates with branched and apolar amino acids. The mutated protein shows no significant changes of S1 to S5 binding pocket specificities. This was assessed by the means of computation and experiments, using a set of substrates with different recognition sites. Also, the stability of Casp‐2 was not affected by either of the mutations, as was tested experimentally using elevated incubation temperatures and chaotropic agents as supplements in the reaction media. Thus, the created mutants are believed to be a first major tool for a toolbox that constitutes an important step toward an universal procedure for *N‐*terminal fusion‐tag cleavage in industrial processes.

## CONFLICT OF INTEREST

The authors declare no conflict of interest.

## Supporting information


**Figure S1** Examplary gels after different times of Casp‐2 cleavage. Only samples showing about 50% cleavage were used for the quantification of the influence of the P1' on cleavage relative to P1' cleavage.
**Figure S2**: Sequence alignment of Casp‐2 and Casp‐3 active site stretches. The numbers given refer to the first amino acid of the following stretch. “|” indicates positions which have a fully conserved residue, “:” indicates conservation between groups of similar properties, equivalent to scoring >0.5 in the Gonnet PAM 250 matrix and “.” indicates conservation between groups of weakly similar properties, equivalent to scoring ≤0.5 in the Gonnet PAM 250 matrix.
**Figure S3**: Experimentally determined time to cleave 50% of the substrate in presence of chaotropic buffer ingredients relative to standard conditions (horizontal line). The substrate VDVAD‐G‐E2 was used for all experiments. Then, 0.1% Tween does not affect the cleavage activities of either of the mutants, compared to the activities under standard conditions. 4 M urea, 1 M GuHCl, and 0.5 M imidazole drop the activities of all mutants and the unmutated protein by roughly two orders of magnitude. However, no significant changes in activity of the mutants towards chaotropic substances compared to the unmutated protein could be detected, indicating unchanged stabilities of the mutants within the tested time of incubation.
**Figure S4**: Experimentally determined time to cleave 50% of the substrate under elevated incubation temperatures compared to the standard activity at 25°C (horizontal line). Both mutants and the unmutated protein show about twice the activity (37°C) or quadruple the activity (50°C). No significant changes in activity of the mutants towards elevated temperatures compared to the unmutated protein could be detected, indicating unchanged stabilities of the mutants within the tested time of incubation.
**Figure S5**: Atoms with atom codes for the tetrahedral intermediate. The ligand (residues 2‐3, Asp‐Ile) was covalently linked to the active site cystein (residue 1). Parameters of the tetrahedral state were generated using the Automated Topology Builder,^1^, ^2^, ^3^ all other parameters were taken from the GROMOS 54A8 parameter set.^4^ See Data S1 for the entire building block.
**Table S1**: Comparison of cleavage entropies for binding pockets S4 to S4' for the proteins Caspase‐2 and Caspase‐3. The difference in cleavage entropy of the S1' binding pocket makes Caspase‐3 an interesting template to enhance the promiscuity of the S1' binding pocket of Caspase‐2.
**Table S2**: FRET substrates (Bachem AG, Germany) used in this study, with P1 position in bold.
**Data S1**: Topology of tetrahedral intermediate.
