# Supplementary material for: Enhancing the promiscuity of a member of the Caspase protease family by rational design
Source: Proteins. 2020 Jun 11;88(10):1303–18. doi: 10.1002/prot.25950 (PMC7497161; doi:10.1002/prot.25950)
Supplement: Supplementary file 1 — Figure S1 Examplary gels after different times of Casp‐2 cleavage. Only samples showing about 50% cleavage were used for the quantification of the influence of the P1' on cleavage relative to P1' cleavage. Figure S2: Sequence alignment of Casp‐2 and Casp‐3 active site stretches. The numbers given refer to the first amino acid of the following stretch. “|” indicates positions which have a fully conserved residue, “:” indicates conservation between groups of similar properties, equivalent to scoring >0.5 in the Gonnet PAM 250 matrix and “.” indicates conservation between groups of weakly similar properties, equivalent to scoring ≤0.5 in the Gonnet PAM 250 matrix. Figure S3: Experimentally determined time to cleave 50% of the substrate in presence of chaotropic buffer ingredients relative to standard conditions (horizontal line). The substrate VDVAD‐G‐E2 was used for all experiments. Then, 0.1% Tween does not affect the cleavage activities of either of the mutants, compared to the activities under standard conditions. 4 M urea, 1 M GuHCl, and 0.5 M imidazole drop the activities of all mutants and the unmutated protein by roughly two orders of magnitude. However, no significant changes in activity of the mutants towards chaotropic substances compared to the unmutated protein could be detected, indicating unchanged stabilities of the mutants within the tested time of incubation. Figure S4: Experimentally determined time to cleave 50% of the substrate under elevated incubation temperatures compared to the standard activity at 25°C (horizontal line). Both mutants and the unmutated protein show about twice the activity (37°C) or quadruple the activity (50°C). No significant changes in activity of the mutants towards elevated temperatures compared to the unmutated protein could be detected, indicating unchanged stabilities of the mutants within the tested time of incubation. Figure S5: Atoms with atom codes for the tetrahedral intermediate. The ligand (residues 2‐3, Asp‐Ile) [file PROT-88-1303-s001.pdf]

Supplementary Material to: Enhancing the promiscuity of a member of the *Caspase* protease family by rational design

Christoph Öhlknecht[1,3], Drazen Petrov[1], Petra Engele[2,3], Christina Krößß[2,3], Bernhard Sprenger[2,3], Andreas Fischer[3], Nico Lingg[3], Rainer Schneider[2], Chris Oostenbrink[1]

[1]Institute of Molecular Modeling and Simulation,
University of Natural Resources and Life Sciences, Vienna, Austria

[2]Institute of Biochemistry and Center of Molecular Biosciences Innsbruck,
University of Innsbruck, Austria

[3] Austrian Centre of Industrial Biotechnology, Muthgasse 18, Vienna, Austria

Table S1: Comparison of cleavage entropies for binding pockets S4-S4' for the proteins Caspase-2 and Caspase-3. The difference in cleavage entropy of the S1' binding pocket makes Caspase-3 an interesting template to enhance the promiscuity of the S1' binding pocket of Caspase-2.

**Prot/Site**	**S4**	**S3**	**S2**	**S1**	**S1'**	**S2'**	**S3'**	**S4'**
**Casp-2**	0.759	0.885	0.909	0.000	0.563	0.921	0.972	0.930
**Casp-3**	0.686	0.919	0.866	0.005	0.835	0.961	0.983	0.969

Table S2: FRET substrates (Bachem AG, Germany) used in this study, with P1 position in bold.

**P1' amino acid**	**Sequence**	**Art.-Nr.**
Alanine	Abz-Val-Asp-Val-Ala-Asp-**Ala**-Ala-Dap(Dnp)-OH	4108736
Glycine	Abz-Val-Asp-Val-Ala-Asp-**Gly**-Ala-Dap(Dnp)-OH	4108741
Isoleucine	Abz-Val-Asp-Val-Ala-Asp-**Ile**-Ala-Dap(Dnp)-OH	4108745
Leucine	Abz-Val-Asp-Val-Ala-Asp-**Leu**-Ala-Dap(Dnp)-OH	4108746
Proline	Abz-Val-Asp-Val-Ala-Asp-**Pro**-Ala-Dap(Dnp)-OH	4108750
Threonine	Abz-Val-Asp-Val-Ala-Asp-**Thr**-Ala-Dap(Dnp)-OH	4108751
Valine	Abz-Val-Asp-Val-Ala-Asp-**Val**-Ala-Dap(Dnp)-OH	4108754

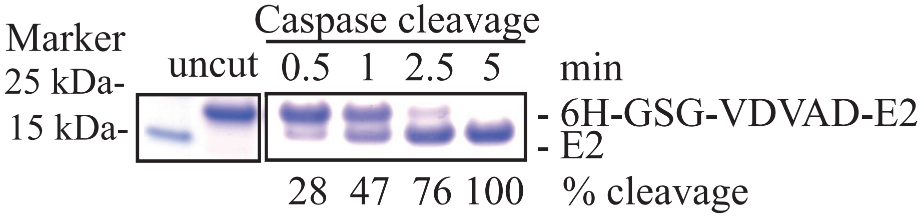

Figure S1: Examplary gels after different times of Casp-2 cleavage. Only samples showing about 50% cleavage were used for the quantification of the influence of the P1' on cleavage relative to P1' cleavage.

```
Casp-2: 212<GEKELEFRSGGDVDH> 273<LLSHGVEGAIYG> 318<QACRGDET>
            ....:..|||.|||.      |||||.||.|:|      |||||.|.
Casp-3:  57<KSTGMTSRSGTDVDA> 118<LLSHGEEGIIFG> 161<QACRGTEL>
 - - - - - - - - - - - - - - - - - - - - - - - - - - - - - -
Casp-2:  375<AAMRNTKR> 418<EGYAPGTEFHRCK>
             .:.||:|.      |.::....||..|
Casp-3:  204<YSWRNSKD> 248<ESFSFDATFHAKK>
```

Figure S2: Sequence alignment of Casp-2 and Casp-3 active site stretches. The numbers given refer to the first amino acid of the following stretch. '|' indicates positions which have a fully conserved residue, ':' indicates conservation between groups of similar properties, equivalent to scoring > 0.5 in the Gonnet PAM 250 matrix and '.' indicates conservation between groups of weakly similar properties, equivalent to scoring =< 0.5 in the Gonnet PAM 250 matrix.

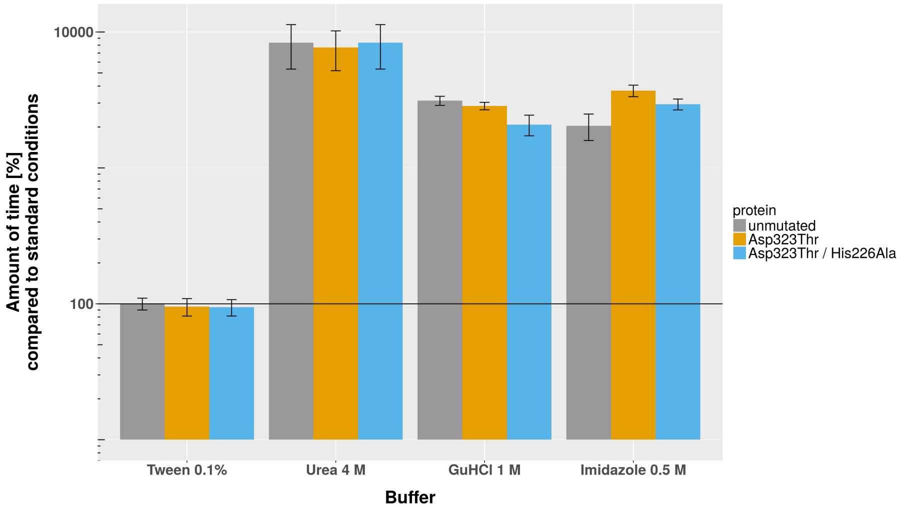

Figure S3: Experimentally determined time to cleave 50% of the substrate in presence of chaotropic buffer ingredients relative to standard conditions (horizontal line). The substrate VDVAD-G-E2 was used for all experiments. 0.1% Tween does not affect the cleavage activities of either of the mutants, compared to the activities under standard conditions. 4 M Urea, 1 M GuHCl and 0.5 M Imidazole drop the activities of all mutants and the unmutated protein by roughly 2 orders of magnitude. However, no significant changes in activity of the mutants towards chaotropic substances compared to the unmutated protein could be detected, indicating unchanged stabilities of the mutants within the tested time of incubation.

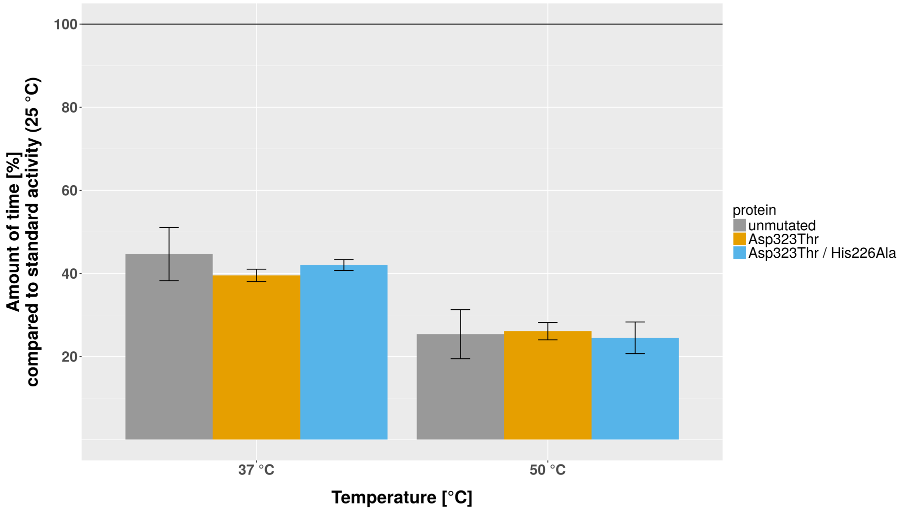

Figure S4: Experimentally determined time to cleave 50% of the substrate under elevated incubation temperatures compared to the standard activity at 25 °C (horizontal line). Both mutants and the unmutated protein show about twice the activity (37 °C) or quadruple the activity (50 °C). No significant changes in activity of the mutants towards elevated temperatures compared to the unmutated protein could be detected, indicating unchanged stabilities of the mutants within the tested time of incubation.

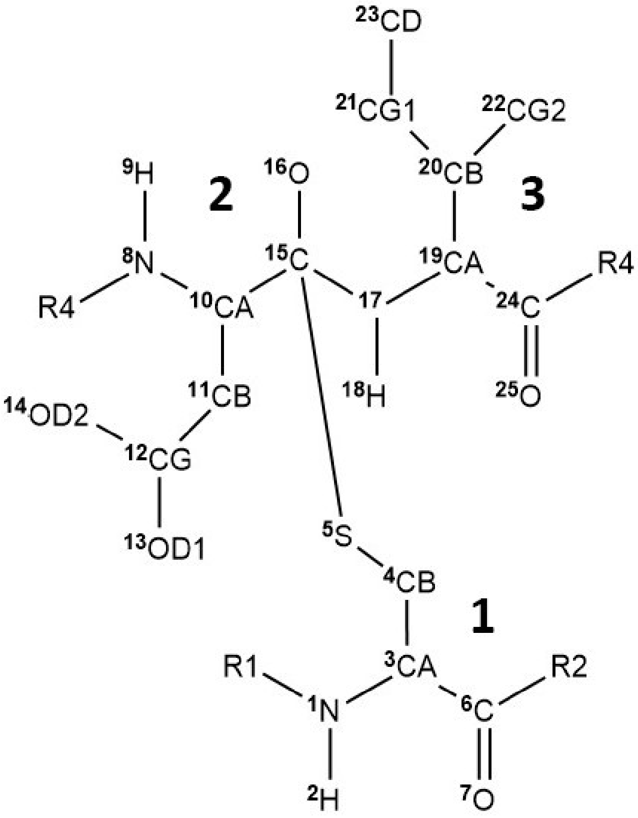

Figure S5: Atoms with atom codes for the tetrahedral intermediate. The ligand (residues 2-3, Asp-Ile) was covalently linked to the active site cystein (residue 1). Parameters of the tetrahedral state were generated using the Automated Topology Builder [1–3], all other parameters were taken from the GROMOS 54A8 parameter set. [4] See supplementary data S1 for the entire building block.

Supplementary Data S1: Topology of tetrahedral intermediate

```
TITLE
Reduced topology using only residues P1, P1' from ligand
and reactive ILE from Casp-2
END
SOLUTEATOM
#   NRP: number of solute atoms
   25
#  ATNM: atom number
#  MRES: residue number
#  PANM: atom name of solute atom
#   IAC: integer (van der Waals) atom type code
#  MASS: mass of solute atom
#    CG: charge of solute atom
#   CGC: charge group code (0 or 1)
#   INE: number of excluded atoms
# INE14: number of 1-4 interactions
# ATNM MRES PANM IAC     MASS       CG  CGC INE
#                                           INE14
     1    1    N   6 14.00670 -0.31000  0     4     2     3     4     6
                                              2     5     7
     2    1    H  21  1.00800  0.31000  1     1     3
                                              2     4     6
     3    1   CA  14 13.01900  0.00000  1     4     4     5     6     7
                                              1    15
     4    1   CB  15 14.02700  0.00000  0     3     5     6    15
                                              4     7    10    16    17
     5    1   SG  23 32.06000 -0.72200  1     4    10    15    16    17
                                              5     6     8    11    18    19
     6    1    C  12 12.01100  0.45000  0     1     7
                                              0
     7    1    O   1 15.99940 -0.45000  1     0
                                              0
     8    2    N   6 14.00670 -0.37000  0     4     9    10    11    15
                                              3    12    16    17
     9    2    H  21  1.00800  0.13200  1     1    10
                                              2    11    15
    10    2   CA  14 13.01900  0.23800  1     5    11    12    15    16    17
                                              4    13    14    18    19
    11    2   CB  15 14.02700  0.16000  0     4    12    13    14    15
                                              2    16    17
    12    2   CG  12 12.01100  0.27000  0     2    13    14
                                              1    15
    13    2  OD1   2 15.99940 -0.71500  0     1    14
                                              0
```

```
    14    2  OD2   2 15.99940 -0.71500  1     0
                                              0
    15    2    C  12 12.01100  0.34600  0     4    16    17    18    19
                                              2    20    24
    16    2    O   1 15.99940 -0.49800  1     1    17
                                              2    18    19
    17    3    N   6 14.00670 -0.32800  0     4    18    19    20    24
                                              3    21    22    25
    18    3    H  21  1.00800  0.20200  1     1    19
                                              2    20    24
    19    3   CA  14 13.01900  0.00000  1     5    20    21    22    24    25
                                              1    23
    20    3   CB  14 13.01900  0.00000  0     4    21    22    23    24
                                              1    25
    21    3  CG1  15 14.02700  0.00000  0     2    22    23
                                              1    24
    22    3  CG2  16 15.03500  0.00000  0     0
                                              2    23    24
    23    3   CD  16 15.03500  0.00000  1     0
                                              0
    24    3    C  12 12.01100  0.45000  0     1    25
                                              0
    25    3    O   1 15.99940 -0.45000  1     0
                                              0
END
BONDSTRETCHTYPE
#  NBTY: number of covalent bond types
252
#  ONLY RELEVANT TYPES ARE SHOWN HERE!
#  CB:  quartic force constant
#  CHB: harmonic force constant
#  B0:  bond length at minimum energy
#    CB               CHB              B0
#2
     1.87000e+07     3.74000e+05     1.00000e-01
#5
     1.66000e+07     5.02283e+05     1.23000e-01
#6
     1.34000e+07     4.18750e+05     1.25000e-01
#10
     1.18000e+07     4.17460e+05     1.33000e-01
#21
     8.71000e+06     3.76429e+05     1.47000e-01
#27
     7.15000e+06     3.34749e+05     1.53000e-01
#32
```

```
     5.62000e+06     3.76416e+05     1.83000e-01
#44
     1.31000e+07     4.19259e+05     1.26500e-01
#240
     7.39645e+03     8.10000e+02     2.34000e-01
END
BOND
#  NBON: number of bonds
24
#  IB, JB: atom sequence numbers of atoms forming a bond
#  ICB: bond type code
#    IB     JB  ICB
      1      2    2
      1      3   21
      3      4   27
      3      6   27
      4      5   32
      5     15  240
      6      7    5
      8      9    2
      8     10   21
     10     11   27
     10     15   27
     11     12   27
     12     13    6
     12     14    6
     15     16   44
     15     17   10
     17     18    2
     17     19   21
     19     20   27
     19     24   27
     20     21   27
     20     22   27
     21     23   27
     24     25    5
END
BONDANGLEBENDTYPE
#  NTTY: number of bond angle types
150
#  ONLY RELEVANT TYPES ARE SHOWN HERE!
#  CT:  force constant (based on potential
#       harmonic in the angle cosine)
#  CHT: force constant (based on potential
#       harmonic in the angle)
#  T0:  bond angle at minimum energy in degrees
```

```
#    CT              CHT             TO
#4
     4.75000e+02     1.40083e-01     1.00000e+02
#11
     4.25000e+02     1.14807e-01     1.09500e+02
#13
     5.20000e+02     1.40521e-01     1.09500e+02
#15
     5.30000e+02     1.40487e-01     1.11000e+02
#16
     5.45000e+02     1.40451e-01     1.13000e+02
#18
     4.60000e+02     1.14885e-01     1.15000e+02
#19
     6.10000e+02     1.52417e-01     1.15000e+02
#22
     6.35000e+02     1.53360e-01     1.17000e+02
#30
     6.85000e+02     1.53127e-01     1.21000e+02
#31
     7.00000e+02     1.53174e-01     1.22000e+02
#33
     7.30000e+02     1.52669e-01     1.24000e+02
#38
     7.70000e+02     1.53365e-01     1.26000e+02
#45
     4.69000e+02     1.40245e-01     9.74000e+01
#46
     5.03000e+02     1.40260e-01     1.06750e+02
#56
     1.68051e+03     4.60000e-01     1.09000e+02
#132
     2.12546e+02     6.40000e-02     8.48000e+01
END
BONDANGLE
#  NTHE: number of bond angles
33
#  IT, JT, KT: atom sequence numbers of atoms
#     forming a bond angle
#  ICT: bond angle type code
#    IT     JT     KT  ICT
      1      3      4   13
      1      3      6   13
      2      1      3   18
      4      3      6   13
      3      4      5   16
```

```
      4      5     15    4
      3      6      7   30
      9      8     10   18
      8     10     11   13
      8     10     15   13
     11     10     15   13
     10     11     12   15
     11     12     13   22
     11     12     14   22
     13     12     14   38
      5     15     10  132
      5     15     16   56
      5     15     17   45
     10     15     16   19
     10     15     17   22
     16     15     17   33
     15     16     17   33
     15     17     18   11
     15     17     19   31
     18     17     19   18
     17     19     20   13
     17     19     24   13
     20     19     24   13
     19     20     21   15
     19     20     22   15
     21     20     22   15
     20     21     23   15
     19     24     25   30
END
IMPDIHEDRALTYPE
#  NQTY: number of improper dihedrals
6
#  ONLY RELEVANT TYPES ARE SHOWN HERE!
#  CQ: force constant of improper dihedral per degrees square
#  Q0: improper dihedral angle at minimum energy in degrees
#           CQ             Q0
#1
    5.10000e-02    0.00000e+00
#2
    1.02000e-01    3.52644e+01
END
IMPDIHEDRAL
#  NQHI: number of improper dihedrals
6
#  IQ,JQ,KQ,LQ: atom sequence numbers of atoms
#    forming an improper dihedral
```

```
#  ICQ: improper dihedral type code
#    IQ     JQ     KQ     LQ  ICQ
      3      1      6      4    2
     10      8     15     11    2
     12     13     14     11    1
     17     15     19     18    1
     19     17     24     20    2
     20     21     22     19    2
END
TORSDIHEDRALTYPE
#  NPTY: number of dihedral types
46
#  ONLY RELEVANT TYPES ARE SHOWN HERE!
#  CP: force constant
#  PD: phase-shift angle
#  NP: multiplicity
#       CP         PD  NP
#14
  33.50000  180.00000   2
#34
   5.92000    0.00000   3
#40
   1.00000    0.00000   6
#42
   3.50000  180.00000   2
#43
   2.80000    0.00000   3
#44
   0.70000  180.00000   6
#45
   0.40000    0.00000   6
#46
   0.00000    0.00000   1
END
DIHEDRAL
#  NPHI: number of dihedrals
12
#  IP, JP, KP, LP: atom sequence numbers
#     of atoms forming a dihedral
#  ICP: dihedral type code
#    IP     JP     KP     LP  ICP
      1      3      4      5   34
      3      4      5     15   46
      4      5     15     17   46
      8     10     11     12   34
      8     10     15     17   42
```

```
      8      10      15      17   45
     10      11      12      13   40
     10      15      17      19   14
     15      17      19      24   43
     15      17      19      24   44
     17      19      20      21   34
     19      20      21      23   34
END
LJPARAMETERS
#  NRATT2: number of LJ interaction types = NRATT*(NRATT+1)/2
1485
#  IAC,JAC: integer (van der Waals) atom type code
#  C12: r**(-12) term in nonbonded interactions
#   C6: r**(-6) term in nonbonded interactions
# CS12: r**(-12) term in 1-4 nonbonded interactions
#  CS6: r**(-6) term in 1-4 nonbonded interactions
# IAC  JAC           C12            C6           CS12            CS6
    1    1  1.000000e-06  2.261954e-03  7.414932e-07  2.261954e-03
#
    1    2  8.611000e-07  2.261954e-03  7.414932e-07  2.261954e-03
    2    2  7.414932e-07  2.261954e-03  7.414932e-07  2.261954e-03
#
    1    6  1.943000e-06  2.347562e-03  1.120291e-06  2.347562e-03
    2    6  3.577063e-06  2.347562e-03  1.120291e-06  2.347562e-03
    6    6  2.319529e-06  2.436410e-03  1.692601e-06  2.436410e-03
#
    1   12  2.222000e-06  2.300953e-03  1.581841e-06  2.300953e-03
    2   12  1.913364e-06  2.300953e-03  1.581841e-06  2.300953e-03
    6   12  3.384106e-06  2.388037e-03  2.389937e-06  2.388037e-03
   12   12  4.937284e-06  2.340624e-03  3.374569e-06  2.340624e-03
#
    1   14  9.850000e-06  3.704924e-03  1.664506e-06  2.566338e-03
    2   14  8.481835e-06  3.704924e-03  1.664506e-06  2.566338e-03
    6   14  1.500155e-05  3.845144e-03  2.514833e-06  2.663466e-03
   12   14  2.188670e-05  3.768802e-03  3.550921e-06  2.610585e-03
   14   14  9.702250e-05  6.068410e-03  3.736489e-06  2.911682e-03
#
    1   15  5.828000e-06  4.110135e-03  1.875128e-06  3.268799e-03
    2   15  5.018491e-06  4.110135e-03  1.875128e-06  3.268799e-03
    6   15  8.876044e-06  4.265691e-03  2.833053e-06  3.392513e-03
   12   15  1.294982e-05  4.181000e-03  4.000245e-06  3.325157e-03
   14   15  5.740580e-05  6.732118e-03  4.209294e-06  3.708671e-03
   15   15  3.396558e-05  7.468416e-03  4.741926e-06  4.723813e-03
#
    1   16  5.162000e-06  4.663258e-03  2.114674e-06  3.937017e-03
    2   16  4.444998e-06  4.663258e-03  2.114674e-06  3.937017e-03
```

```
    6   16  7.861726e-06  4.839748e-03  3.194972e-06  4.086021e-03
   12   16  1.146996e-05  4.743659e-03  4.511272e-06  4.004896e-03
   14   16  5.084570e-05  7.638095e-03  4.747027e-06  4.466809e-03
   15   16  3.008414e-05  8.473481e-03  5.347702e-06  5.689469e-03
   16   16  2.664624e-05  9.613802e-03  6.030865e-06  6.852528e-03
#
    1   21  0.000000e+00  0.000000e+00  0.000000e+00  0.000000e+00
    2   21  0.000000e+00  0.000000e+00  0.000000e+00  0.000000e+00
    6   21  0.000000e+00  0.000000e+00  0.000000e+00  0.000000e+00
   12   21  0.000000e+00  0.000000e+00  0.000000e+00  0.000000e+00
   14   21  0.000000e+00  0.000000e+00  0.000000e+00  0.000000e+00
   15   21  0.000000e+00  0.000000e+00  0.000000e+00  0.000000e+00
   16   21  0.000000e+00  0.000000e+00  0.000000e+00  0.000000e+00
   21   21  0.000000e+00  0.000000e+00  0.000000e+00  0.000000e+00
#
    1   23  3.616000e-06  4.752195e-03  3.113738e-06  4.752195e-03
    2   23  3.113738e-06  4.752195e-03  3.113738e-06  4.752195e-03
    6   23  5.507168e-06  4.932051e-03  4.704416e-06  4.932051e-03
   12   23  8.034752e-06  4.834130e-03  6.642592e-06  4.834130e-03
   14   23  3.561760e-05  7.783768e-03  6.989728e-06  5.391683e-03
   15   23  2.107405e-05  8.635086e-03  7.874189e-06  6.867502e-03
   16   23  1.866579e-05  9.797156e-03  8.880108e-06  8.271378e-03
   21   23  0.000000e+00  0.000000e+00  0.000000e+00  0.000000e+00
   23   23  1.307546e-05  9.984006e-03  1.307546e-05  9.984006e-03
END
```

References

[1] Malde AK, Zuo L, Breeze M, et al. An Automated Force Field Topology Builder (ATB) and Repository: Version 1.0 J. Chem. Theory Comput. 2011;7:4026-4037.

[2] Canzar S, El-Kebir M, Pool R, et al. Charge Group Partitioning in Biomolecular Simulation J. Comput. Biol. 2013;20:188-198.

[3] Koziara KB, Stroet M, Malde AK, Mark AE. Testing and validation of the Automated Topology Builder (ATB) version 2.0: prediction of hydration free enthalpies J. Comput. Aid. Mol. Des. 2014;28:221–233.

[4] Reif MM, Hünenberger PH, Oostenbrink C. New Interaction Parameters for Charged Amino Acid Side Chains in the GROMOS Force Field J. Chem. Theory Comput. 2012;8(10):3705-3723.
